# The Amino Acid Transporter OsAAP4 Contributes to Rice Tillering and Grain Yield by Regulating Neutral Amino Acid Allocation through Two Splicing Variants

**DOI:** 10.1186/s12284-020-00446-9

**Published:** 2021-01-06

**Authors:** Zhongming Fang, Bowen Wu, Yuanyuan Ji

**Affiliations:** 1 Key Laboratory of Plant Resource Conservation and Germplasm Innovation in Mountainous Region (Ministry of Education), College of Agricultural Sciences, Guizhou University, Guiyang, 550025 China; 2 National Key Laboratory of Crop Genetic Improvement, Huazhong Agricultural University, Wuhan, 430070 China

**Keywords:** Amino acid, Transporter, Rice, Tiller, Outgrowth bud, Grain yield, Splicing variants

## Abstract

**Background:**

Amino acids, which are transported by amino acid transporters, are the major forms of organic nitrogen utilized by higher plants. Among the 19 Amino Acid Permease transporters (AAPs) in rice, only a small number of these genes have been reported to influence rice growth and development. However, whether other OsAAPs are responsible for rice growth and development is unclear.

**Results:**

In this study, we demonstrate that *OsAAP4* promoter sequences are divergent between *Indica* and *Japonica*, with higher expression in the former, which produces more tillers and higher grain yield than does *Japonica*. Overexpression of two different splicing variants of *OsAAP4* in *Japonica* ZH11 significantly increased rice tillering and grain yield as result of enhancing the neutral amino acid concentrations of Val, Pro, Thr and Leu. *OsAAP4* RNA interference (RNAi) and mutant lines displayed opposite trends compared with overexpresing (OE) lines. In addition, exogenous Val or Pro at 0.5 mM significantly promoted the bud outgrowth of lines overexpressing an *OsAAP4a* splicing variant compared with ZH11, and exogenous Val or Pro at 2.0 mM significantly enhanced the bud outgrowth of lines overexpressing splicing variant *OsAAP4b* compared with ZH11. Of note, the results of a protoplast amino acid-uptake assay showed that Val or Pro at different concentrations was specifically transported and accumulated in these overexpressing lines. Transcriptome analysis further demonstrated that OsAAP4 may affect nitrogen transport and metabolism, and auxin, cytokinin signaling in regulating rice tillering.

**Conclusion:**

Our results suggested that *OsAAP4* contributes to rice tiller and grain yield by regulating neutral amino acid allocation through two different splicing variants and that *OsAAP4* might have potential applications in rice breeding.

**Supplementary Information:**

The online version contains supplementary material available at 10.1186/s12284-020-00446-9.

## Background

Nitrogen is one of the limiting nutrients for plant growth and development. Higher plants take up inorganic nitrogen, including nitrate and ammonium; this is followed by nitrogen assimilation into amino acids, the main form of organic nitrogen transport, in the root and transport and reallocation from source organs to sinks via the xylem and phloem (Xu et al. 2012; Tegeder and Masclaux-Daubresse, 2018). Plants also acquire amino acids directly from the soil (Tegeder and Rentsch, 2010). Amino acids are the main components of the enzymes and proteins involved in plant metabolism and structure and also serve as precursors for the synthesis of a large variety of compounds critical to plant development, including nucleotides, chlorophyll, and secondary metabolites such as hormones and lignin (Tegeder, 2012; Pratelli and Pilot, 2014; Jin et al. 2019). Amino acid transporters play an important role in the transmembrane transport of amino acids, which are involved directly or indirectly in processes of nitrogen metabolism that are crucial for plant growth and development. Such processes include assimilation and partition of amino acids within the cell, translocation of amino acids over short and long distances, and uptake and usage of amino acids by sink organs (Tegeder, 2014; Tegeder and Masclaux-Daubresse, 2018). Recent studies have shown that increasing phloem and embryo loading with amino acids may increase biomass and seed yield (Zhang et al. 2015; Perchlik and Tegeder, 2017; Tegeder and Masclaux-Daubresse, 2018).

Amino acid permease (AAP), a member of the amino acid transporter (AAT) family, has been extensively studied functionally in plants. AAPs have been suggested to be involved in a number of physiological processes in plants, including amino acid uptake from the soil, phloem loading or xylem-phloem transfer, and seed loading (Tegeder and Rentsch 2010). In *Arabidopsis thaliana*, 8 AAP transporters (AtAAP1-AtAAP8) are reported to have important functions in the translocation of different amino acids for organic nitrogen utilization in source and sink organs. For example, it has been demonstrated that AtAAP1 imports neutral, uncharged amino acids into root cells and developing embryos and is important for storage protein synthesis and seed yield in *Arabidopsis* (Hirner et al. 1998; Lee et al. 2007; Sanders et al. 2009). AtAAP2 was found to transport Glu and neutral amino acids and be very important for amino acid transport from the xylem to phloem (Fischer et al. 2002; Zhang et al. 2010). In addition, AtAAP3 mediates the uptake of neutral and basic amino acids (Okumoto et al. 2004), AtAAP4 imports neutral amino acids Pro and Val (Fischer et al. 1995), and broad-affinity AtAAP5 transports anionic, neutral and cationic amino acids (Fischer et al. 1995; Boorer and Fischer, 1997; Svennerstam et al. 2008). AtAAP6 reportedly affects the Lys, Phe, Leu and Asp contents of sieve elements and regulates rosette width and seed volume in *Arabidopsis* (Hunt et al. 2010), and AtAAP8, a high-affinity transporter of acidic amino acids, is important for seed development and yield (Okumoto et al. 2002; Schmidt et al. 2007; Santiago and Tegeder, 2016).

In *Vicia faba*, VfAAP1 and VfAAP3 transport a broad range of amino acids, though VfAAP1 has a preference for Cys and VfAAP3 for Lys and Arg (Miranda et al. 2001). *StAAP1* is expressed in mature leaves, and antisense inhibition of this gene decreases the amino acid content of transgenic *Solanum tuberosum* (Koch et al. 2003). *PvAAP1* is expressed in epidermal cells, xylem parenchyma cells, and phloem and is involved in xylem-phloem transfer and phloem loading for amino acid transport to sink tissues in *Phaseolus vulgaris* (Tan et al. 2008). It was also proposed that PtAAP11 plays a major role in xylem formation by providing Pro in *Populus trichocarpa* (Couturier et al. 2010). Recently, it was found that overexpression of *PsAAP1* positively regulated amino acid transport from source to sink organs and influenced plant nitrogen use efficiency in *Pisum sativum* (Perchlik et al. 2017), and PsAAP6 functions in nodule nitrogen metabolism and export and plant nutrition (Garneau et al. 2018).

Among rice 19 AAP transporters in rice, OsAAP6 was reported to affect the distribution of various amino acids in plants and to function as a positive regulator of the grain protein content and grain quality in rice (Peng et al. 2014). OsAAP3 mainly transports basic amino acids Lys and Arg (Taylor et al. 2015), and recent studies demonstrated that blocking *OsAAP3* or *OsAAP5* expression increases grain yield by regulating the concentrations of these two amino acids (Lu et al. 2018; Wang et al. 2019a). Moreover, the amino acid transporter OsAAP1 mediate growth and grain yield by regulating neutral amino acid uptake and reallocation in rice (Ji et al. 2020). In rice, in addition to OsAAPs, OsLHT1 has been shown to function in amino acid root uptake and source-to-sink allocation (Liu et al. 2005; Wang et al. 2019b; Guo et al. 2020a, 2020b). In this study, we found the promoter sequences of *OsAAP4* to be divergent between *Indica* and *Japonica*, resulting in higher expression of *OsAAP4* in *Indica,* which produced more tillers and higher grain yield than did *Japonica*. Moreover, two variants of OsAAP4 mainly regulated neutral amino acid Val and Pro within different concentration ranges and significantly increased grain yield by promoting bud outgrowth and increasing tiller number. *OsAAP4* might have potential applications in rice breeding to increase grain yield especially in plants grown in soil with abundant organic nitrogen.

## Results

### The Expression Level of *OsAAP4* Positively Correlated with Rice Tillering and Grain Yield between *Indica* and *Japonica*

**Fig. 1:**
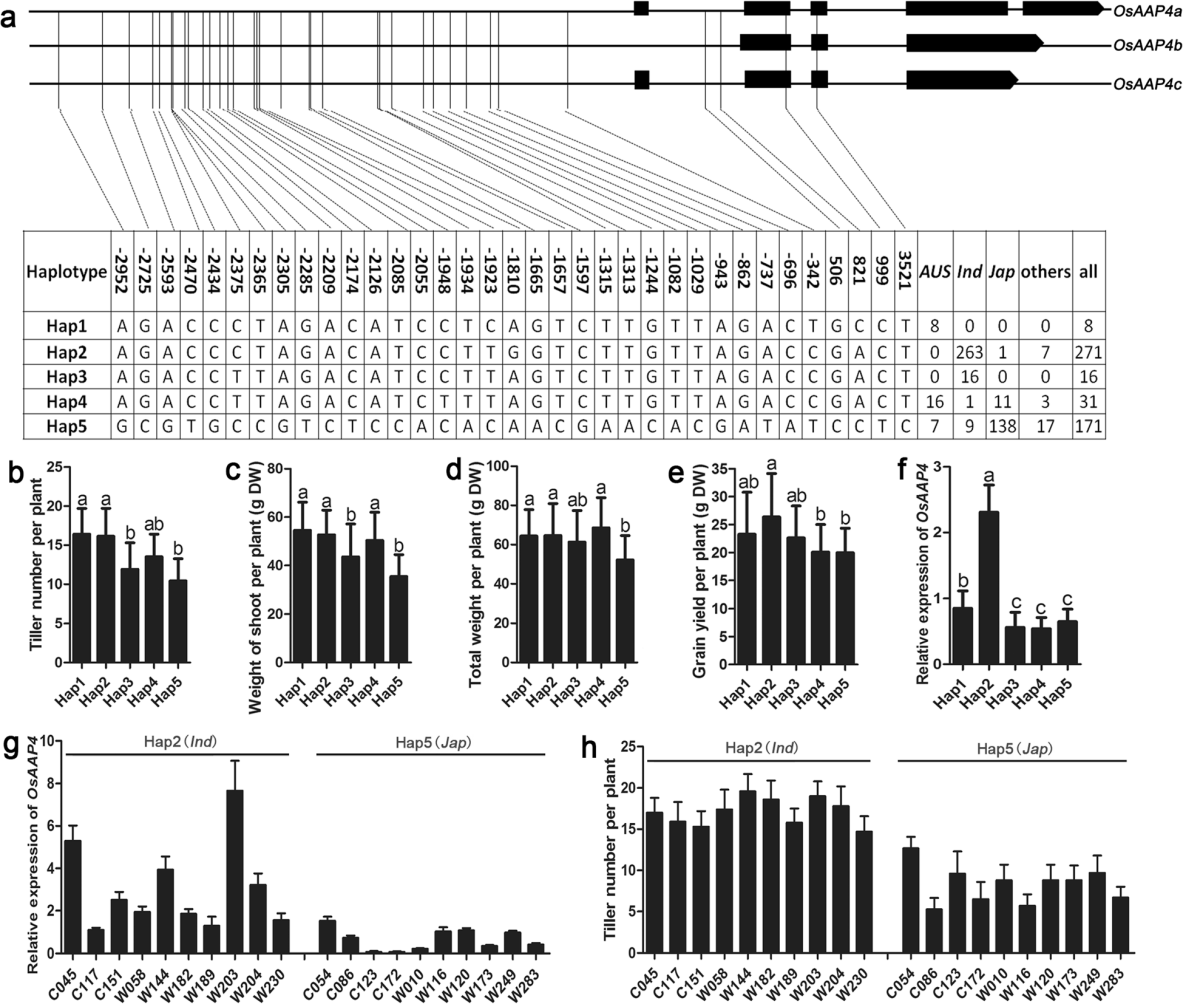
The expression level of *OsAAP4* was positively correlated with rice growth between *Indica* and *Japonica.* **a** SNP divergence in *OsAAP4* promoter regions between rice *Indica* and *Japonica*. **b** Average tiller number per plant at filling stage in *OsAAP4* haplotypes 1 to 5 (Hap1-Hap5). **c** Average weight of shoot per plant at filling stage in *OsAAP4* Hap1-Hap5. **d** Average total weight per plant at mature stage in *OsAAP4* Hap1-Hap5. **e** Average grain yield per plant at mature stage in *OsAAP4* Hap1-Hap5. **f** Average expression levels of *OsAAP4* in young seedling tiller bud of Hap1-Hap5. **g** Expression levels of *OsAAP4* in young seedling tiller bud between Hap2 and Hap5 of ten individual varieties. **h** Tiller number of ten individual varieties between Hap2 and Hap5. Four hundred ninety-seven rice accessions with Hap1-Hap5 according to Rice Variation Map v2.0 were used in (**b**-**f**). The primers used for quantifying *OsAAP4* expression (**f**, **g**) was F: GACATCGTCCACAACCTCAAGGCT, and R: GCCACAGCTCTAGCTAGGCAGC. The letters above the error bars are ranked by the Duncan test at *p* < 0.05. Values are means ± s.d. (*n* = 3)

Overall, 533 rice accessions according to Rice Variation Map v2.0 (a database for rice genome variation) were used in this study (Chen et al. 2014). First, we analyzed the promoter and exon sequences of *OsAAP4* in all 533 accessions and identified 5 haplotypes in 497 accessions (Fig. 1a). Among these materials, 35 single-nucleotide polymorphisms (SNPs) were detected in haplotypes 1 to 5 (Hap1-Hap5) (Fig. 1a). Surprisingly, Hap2 was found to be mainly present in *Indica* accessions, whereas was Hap5 mainly found in *Japonica* accessions (Fig. 1a). These results indicate various divergences of *OsAAP4* promoter sequences between *Indica* and *Japonica*. We then detected tiller number per plant (Fig. 1b) and weight of shoot per plant (Fig. 1c) in the aboveground parts at filling stage, total weight per plant (Fig. 1d) and grain yield per plant (Fig. 1e) at mature stage of Hap1 to Hap5-type cultivar seedlings and found that the tiller number, weight of shoot, total weight, grain yield in *Indica* (Hap2) was significantly higher than that in *Japonica* (Hap5, Fig. 1b-e). Furthermore, We chose seedlings at the vegetative stage to detect expression of *OsAAP4* from Hap1 to Hap5 and found that *OsAAP4* expression in *Indica* accessions (Hap2) was significantly higher than that in *Japonica* cultivars (Hap5, Fig. 1f). In addition, we randomly selected ten *Indica* and ten *Japonica* cultivars to assess the association of *OsAAP4* expression level with tiller number in seedlings of different Haps and found that the expression levels of *OsAAP4* in the *Indica* cluster with Hap2 were higher than those in the *Japonica* cluster with Hap5 (Fig. 1g). Moreover, the expression levels of *OsAAP4* in Hap2-*Indica* accessions were higher than those in Hap5-*Japonica* accessions at basal part of seedlings (Supplementary file 1: Figure S1b). However, no difference of *OsAAP4* expression levels bewteen Hap2 and Hap5 accessions was observed at root, old leaf, and young leaf of seedlings (Supplementary file 1: Figure S1a, c-d). Similarly, tiller number per plant were higher in seedlings of *Indica* accessions that carried Hap2 compared to *Japonica* accessions carrying Hap5 (Fig. 1h). These results demonstrated that *Indica* accessions with Hap2 more highly expressed *OsAAP4*, which was accompanied by higher tiller numbers and grain yield, than *Japonica* accessions, indicating that *OsAAP4* expression levels are positively correlated with both tiller development and grain yield in rice.

### The Expression Pattern of *OsAAP4* and Subcellular Localization of the Protein

**Fig. 2:**
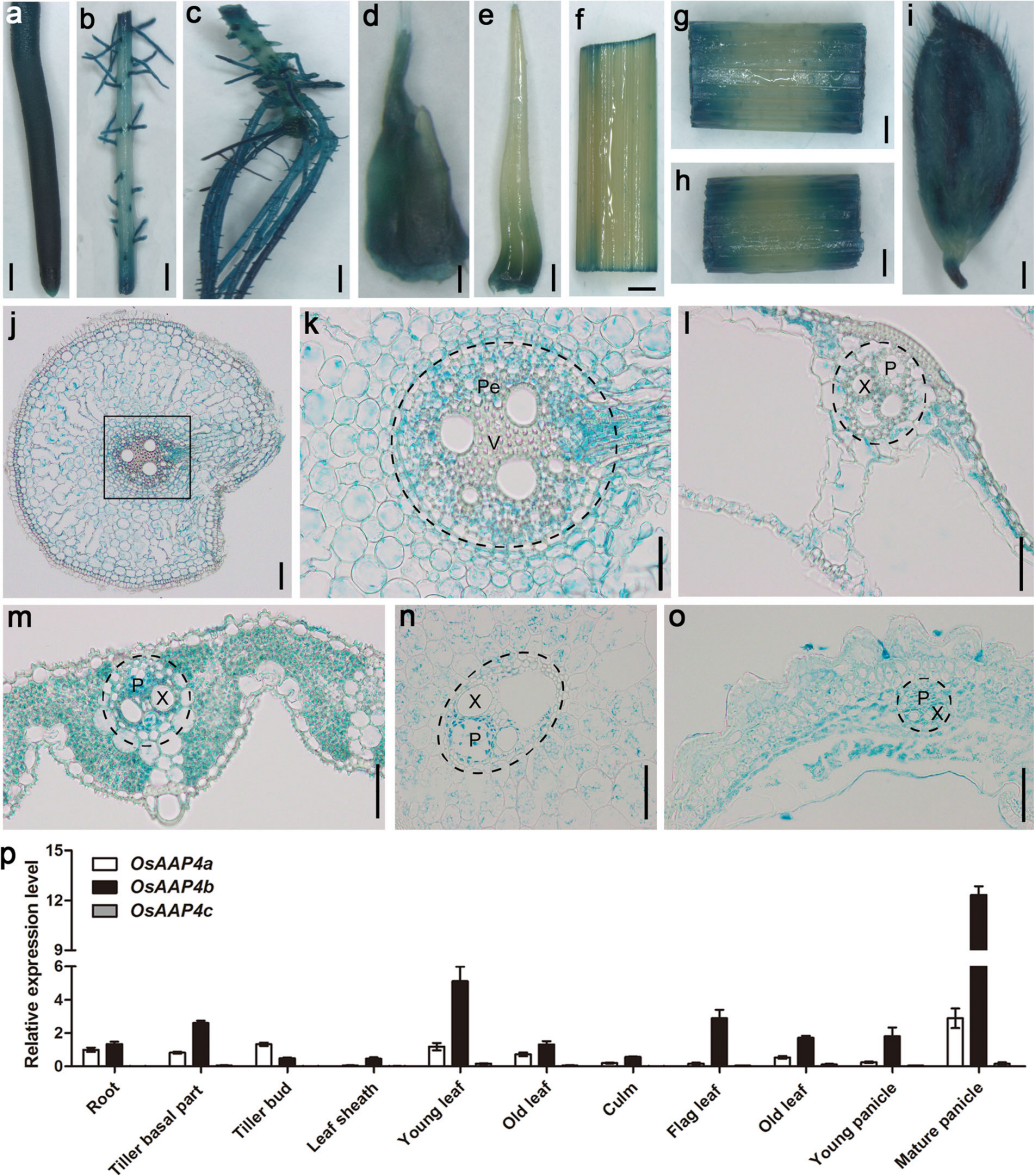
Promoter-GUS analysis and relative expression level of *OsAAP4. OsAAP4* promoter**-**GUS staining in the root tip (**a**), lateral root (**b**), adventitious roots (**c**), short outgrowth bud (**d**), long outgrowth bud (**e**), leaf blade **(f**), leaf sheath (**g**), stem (**h**), and panicle (**i**) using the Hap2-*Indica* type of p*OsAAP4*-*GUS-*transgenic plants. Transverse section of a root (**j**) and its enlargement (**k**), leaf sheath (**l**), leaf blade (**m**), stem (**n**), and panicle (**o**) using the Hap2-*Indica* type of p*OsAAP4-GUS* transgenic plants. **p** The expression pattern of *OsAAP4* in different tissues of *Japonica* ZH11. The primers used for quantifying *OsAAP4a* expression was F: TGGCACTCACCCTTGCACAC, and R: CCGTCCACACCGTCCCTTGT, for quantifying *OsAAP4b* expression was ACTTGAGCTCTCTGCATTGGGT, and R: AGCGGTAGCAATTGGCGAGGA, and for quantifying *OsAAP4b + c* expression was TTGCTGCAGGTGTTCGCGCA, ATCGTCCGCAGCACCAGCTTCAG which primers were designed for common sequences between two splice variants *OsAAP4b* and *OsAAP4c*. *OsAAP4c* of the two splice variants accounts for half of the expression level for both the splice variants. Pe indicates pericycle, V indicates vascular, X indicates xylem, and P indicates phloem in (**k**-**o**). Scale bars, 0.5 cm (**a**-**c**, **f**-**h**), 0.2 cm (**d**), 0.1 cm (**e**, **i**), 50.0 μm (**j**), 20.0 μm (**k**-**o**)

To further compare *OsAAP4* promoter activity between Hap2 and Hap5, we amplified promoter sequences by PCR and performed sequencing (Supplementary file 2: Figure S2). The results showed that the promoter sequence of the Hap5 type in *Japonica* was the same as that of *Japonica* Nipponbare, which has been sequenced (https://phytozome.jgi.doe.gov/pz/portal.html). However, there were many SNP differences in the sequence of Hap2 type in *Indica*, with also base insertion and deletion in the promoter region of Hap2 compared with Hap5 (Supplementary file 2: Figure S2). Therefore, a promoter-GUS plasmid of Hap2 type of *OsAAP4* was constructed and transformed into *Japonica* ZH11. GUS staining revealed a particularly strong signal in the root tip (Fig. 2a), lateral root (Fig. 2b, c), and young tiller bud (Fig. 2d) at the vegetative stage and the leaf blade (Fig. 2f), leaf sheath (Fig. 2g), stem (Fig. 2h) and panicle (Fig. 2i) at the reproductive stage. Additionally, GUS activity was abundant in the parenchymal cells of the cortex in a transverse section of the root (Fig. 2j, k) and was enriched in the xylem and phloem of vascular tissue in the leaf sheath (Fig. 2l), leaf blade (Fig. 2m), stem (Fig. 2n), and young panicle (Fig. 2o).

Next, we detected the levels of three splicing variants for the *OsAAP4* gene in various tissues. Expression levels of the longest variant *OsAAP4a* were higher in the root, tiller basal part, tiller bud, and leaf at the vegetative stage in *Japonica* ZH11, but the levels of the moderate-length variant *OsAAP4b* were higher in the root, tiller basal part, and leaf at the vegetative stage and the leaf and panicle at the reproductive stage (Fig. 2p). The expression level of the shortest splicing variant *OsAAP4c* was lower in various tissues (Fig. 2p). Besides, the expression levels of *OsAAP4a* or *OsAAP4b* in the basal part for tiller bud elongation in hap2 varieties were higher than those in hap5 varieties (Supplementary file 3: Figure S3). We also observed enrichment of green fluorescence signals of OsAAP4a-GFP and OsAAP4b-GFP both in the plasma membrane and the nucleus (Supplementary file 4: Figure S4). These results indicated that OsAAP4 more likely mediates amino acid membrane allocation from roots through parenchymal cells and reallocates amino acids from source organs to sinks via the xylem and phloem.

### *OsAAP4* Positively Regulated Rice Tillering and Grain Yield

**Fig. 3:**
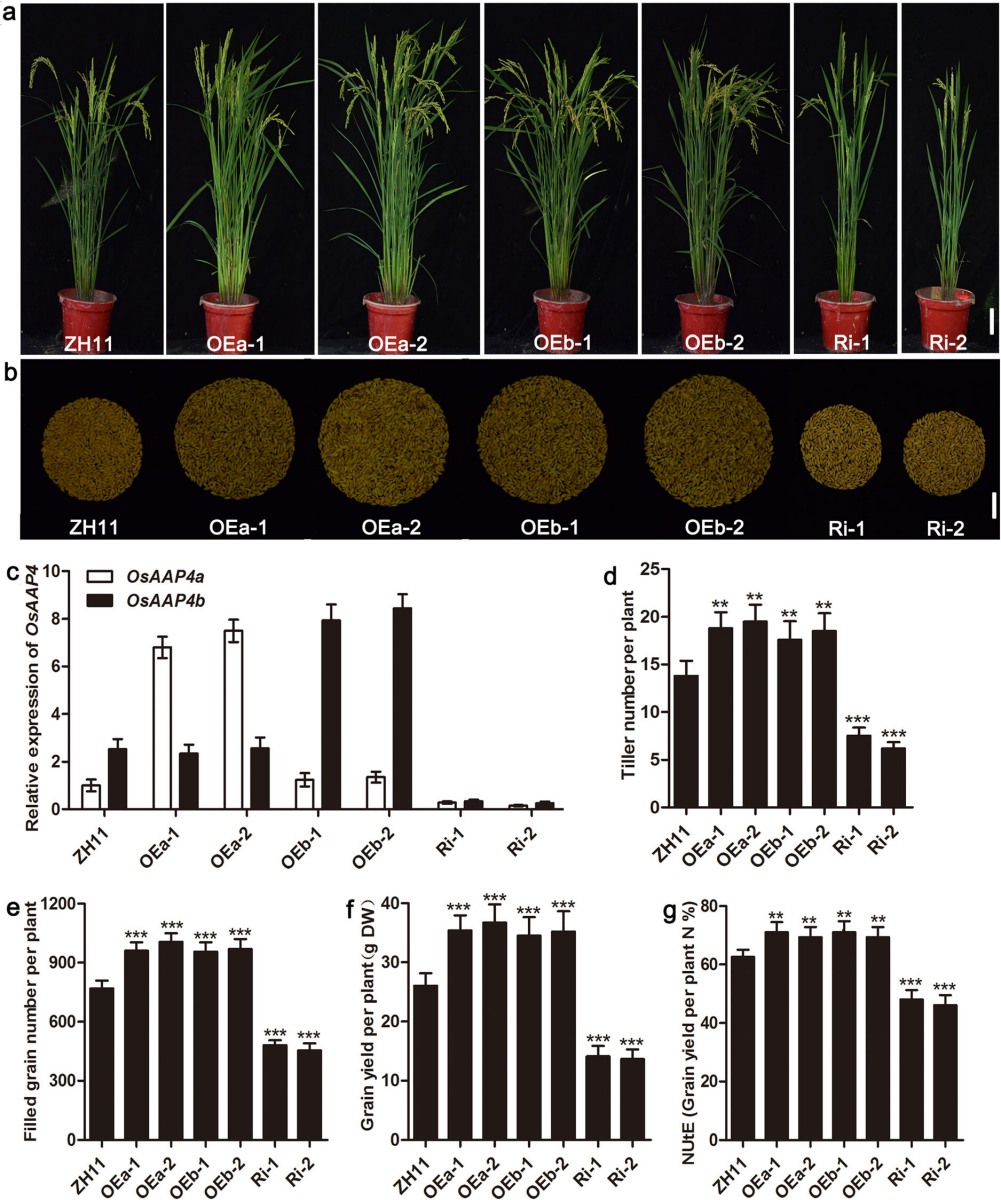
Phenotypic analysis of *OsAAP4* transgenic plants in the *Japonica* ZH11 background grown in Wuhan paddy fields. Whole-plant phenotype (**a**), grain yield phenotype (**b**), relative expression of *OsAAP4* in the leaf blade (**c**), tiller number per plant (**d**), filled grain number per plant (**e**), grain yield per plant (**f**) and nitrogen utilization efficiency (NUtE) of transgenic plants and ZH11 (**g**). OEa-1 and OEa-2 indicate long variants of *OsAAP4a-*overexpressing lines, OEb-1 and OEb-2 indicate short variants *OsAAP4b-*overexpressing lines, and Ri-1 and Ri-2 indicate *OsAAP4*-RNAi lines. The primers used for quantifying *OsAAP4a* expression was F: TGGCACTCACCCTTGCACAC, and R: CCGTCCACACCGTCCCTTGT, for quantifying *OsAAP4b* expression was ACTTGAGCTCTCTGCATTGGGT, and R: AGCGGTAGCAATTGGCGAGGA. The letters above the error bars are ranked by the T test, “**” indicates a significant difference at *p* < 0.01, and “***” indicates a significant difference at *p* < 0.001. Scale bar, 10.0 cm (**a**), 2.0 cm (**b**). Values are means ± s.d. (*n* > 20)

To further understand the effects of altered *OsAAP4* expression on rice growth and development, we generated longer variant OEa (over-expression), shorter variant OEb (over-expression) and Ri (common sequence of the two variants of RNAi) transgenic lines of *OsAAP4* under the control of rice 35S and *Ubi-1* promoters. Compared with wild-type ZH11, OEa and OEb lines showed significantly higher tiller numbers at the reproductive stage, whereas the two Ri lines exhibited reduced numbers of tiller (Fig. 3a, d). Moreover, we detected the expression levels of *OsAAP4* in the transgenic plants and found that the OEa and OEb lines for each variant showed significantly higher expression levels than did wild-type ZH11 but that the Ri lines showed markedly reduced levels of *OsAAP4* expression than ZH11 (Fig. 3c). In addition, overexpression of *OsAAP4* in OEa and OEb lines resulted in enhanced filled grain number and grain yield per plant compared with ZH11 (Fig. 3e, f). More importantly, nitrogen utilization efficiency (NUtE) was significantly improved in *OsAAP4* OEa and OEb lines compared with ZH11; however, Ri lines showed reduced NUtE than ZH11 (Fig. 3g). To further investigate the impact of *OsAAP4* on rice growth and development, we established a CRISPR line of the common sequence of the two variants of *OsAAP4* (Supplementary file 5: Figure S5) and found that *OsAAP4* knockout significantly decreased tiller number (Supplementary file 5: Figure S5b, d), filled grain number (Supplementary file 5: Figure S5c, e), grain yield (Supplementary file 5: Figure S5c, f), and NUtE (Supplementary file 5: Figure S5g) compared to ZH11. Similarly, tiller number per plant were higher in OEa and OEb lines compared to ZH11 at the reproductive stage, whereas the two Ri lines and *osaap4* exhibited reduced numbers of tiller when rice plants were grown in Sanya, China (Supplementary file 6: Figure S6).

### Two Variants of *OsAAP4* OE Lines Promoted Bud Outgrowth under Different Neutral Amino Acid Concentrations

**Fig. 4:**
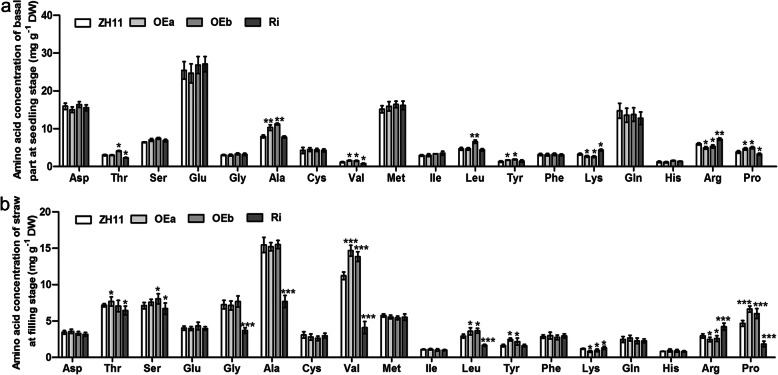
Effect of *OsAAP4* on amino acid concentrations among ZH11, OEa, OEb, and Ri lines. Amino acid concentrations of basal parts at seedlings stage (**a**) and straw at filling stage (**b**). OEa, OEb, and Ri indicated that mixed equal-amount which extracted from each three OEa, OEb, and Ri lines, respectively. The letters above the error bars are ranked by the T test, “*” indicates a significant difference at *p* < 0.05, “**” indicates a significant difference at *p* < 0.01, and “***” indicates a significant difference at *p* < 0.001. Values are means ± s.d. (*n* = 3)

To further investigate the amino acids accompanying enhanced expression levels of *OsAAP4* in rice growth and development, we measured the concentration of individual amino acids in the basal parts at seedling stage and straws at aboveground parts of transgenic plants. The results showed that concentrations of neutral amino acids Thr, Val, Leu, Tyr, and Pro were higher in OEa or OEb than in ZH11, however, the concentrations of basic amino acids Lys and Arg were significantly decreased when compared with levels in ZH11 (Fig. 4). In contrast, the concentrations of neutral amino acids Thr, Val, and Pro in Ri line seedlings were significantly decreased compared with those of ZH11 (Fig. 4). Moreover, accumulation of basic amino acids Lys and Arg was found in Ri line seedlings compared to ZH11 (Fig. 4). In addition, the concentrations of total amino acids were higher in OEa and OEb compared with ZH11, but lower concentrations of total amino acids in Ri (Supplementary file 7-Figure S7). These results indicated that the concentrations of Val and Pro increased most significantly in the OE line and decreased in the Ri line, suggesting that overexpression of *OsAAP4* might promote the allocation of neutral amino acids Val and Pro to further support plant growth and enhance grain yield. However, Ri lines suppressing *OsAAP4* showed decreased contents of neutral amino acids Val and Pro and enhanced contents of basic amino acids Lys and Arg to balance the total amino acid content in seedlings.

**Fig. 5:**
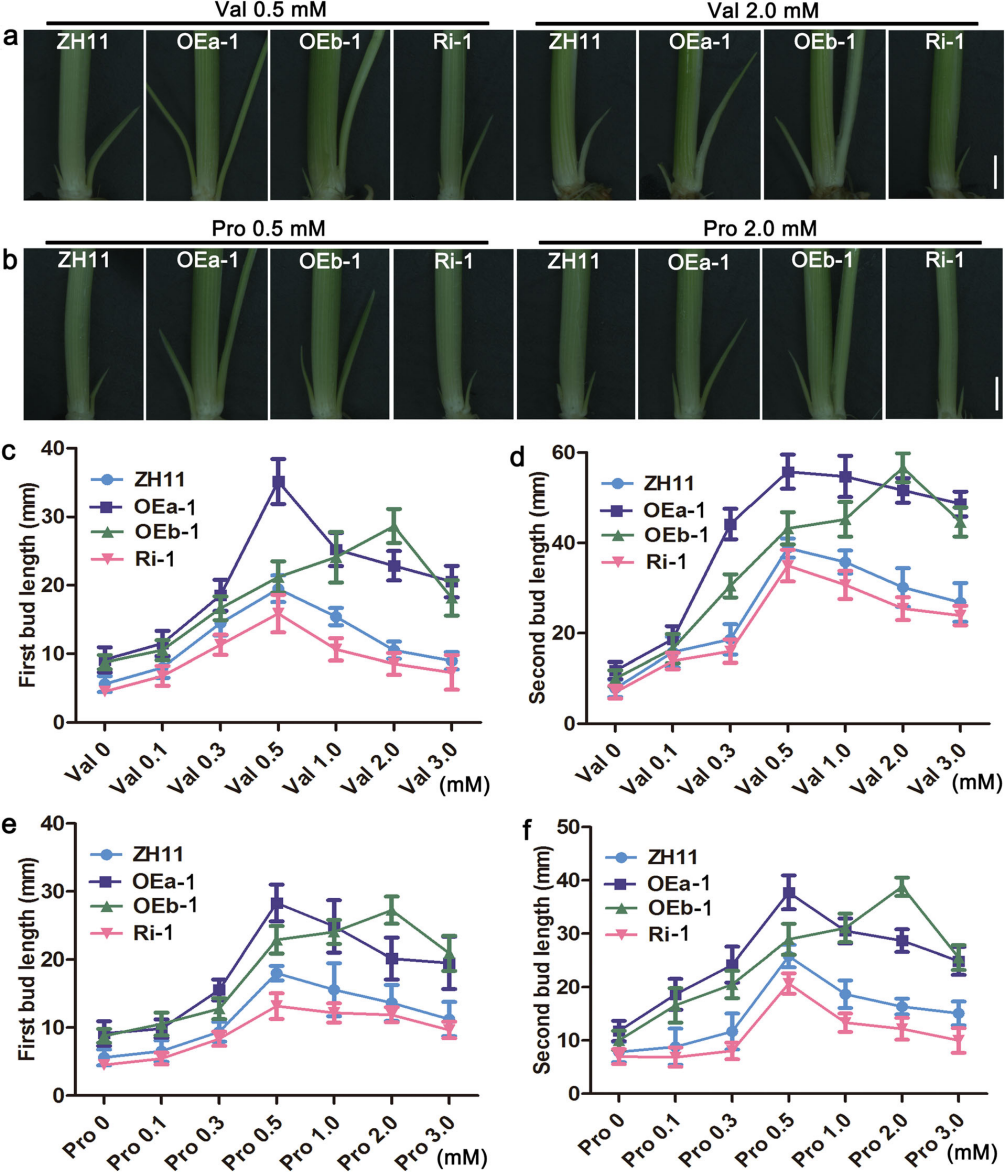
Effect of different concentrations of Val and Pro on bud outgrowth among ZH11, OEa, OEb, and Ri lines grown in hydroponic culture. Phenotypes of outgrowth buds among ZH11, OEa, OEb, and Ri lines grown with 1.0 mM NH_4_NO_3_ and 0.5 mM Val and 2.0 mM (**a**), 0.5 mM Pro and 2.0 mM Pro (**b**). Quantification of the first bud (**c**) and second bud (**d**) under different concentrations of Val. Quantification of the first bud (**e**) and second bud (**f**) under different concentrations of Pro. Scale bars, 1.0 cm (**a**, **b**). Values are means ± s.d. (*n* > 15)

As the number of tillers in OE lines increased at the reproductive stage compared to that in ZH11 (Fig. 3), we further validate the effect of Val and Pro on bud outgrowth for tillering among different *OsAAP4* expression lines, exogenous Val and Pro was applied. Interestingly, both the first bud and second bud length of OEa line increased under the 0.5 mM Val treatment compared with OEb line. However, the first bud and second bud length in OEb line increased when the concentration of Val was 2.0 mM compared with OEa line (Fig. 5a, c, d), and results similar those for Val were observed at 0.5 or 2.0 mM Pro (Fig. 5b, e, f). Additionally, the first and second bud lengths of the Ri line decreased when compared with ZH11 at these concentrations of amino acids treatments (Fig. 5). Besides, the plant height and biomass of OEa and OEb lines were notably increased compared with that of wild-type ZH11 under Val 0.5 mM treatment after 6 weeks (Supplementary file 8: Figure S8a, e, f), but these aspects were significantly reduced compared with ZH11 under Val 2.0 mM treatment (Supplementary file 8: Figure S8b, e, f). In addition, 0.5 mM Pro strongly increased plant height and biomass only in OEa plants (Supplementary file 8: Figure S8c, g, h) and 2.0 mM Pro significantly promoted plant height and biomass in OEb plants (Supplementary file 8: Figure S8d, g, h) compared with ZH11 after 6 weeks. No obvious effect on plant height and biomass of the *OsAAP4* Ri lines compared with ZH11 was found for 0.5 mM Pro treatment (Supplementary file 8: Figure S8g, h), but 2.0 mM Pro significantly decreased the biomass of Ri lines compared with ZH11 (Supplementary file 8: Figure S8h). Analysis of bud outgrowth, plant height and biomass revealed that elevated expression of *OsAAP4a* facilitates rice tillering at lower concentrations of Val and Pro (0.5 mM) but that *OsAAP4b* facilitates rice tillering at higher amino acid concentrations of Val and Pro (2.0 mM). Interestingly, the two splicing variants displayed different sensitivities to different amino acid concentrations. Taken together, these results demonstrated that two OE line variants promoted rice tillering under different concentrations of Val and Pro.

### Both Variants of OsAAP4 Might Regulate Neutral Amino Acids to Support Rice Tillering

**Fig. 6:**
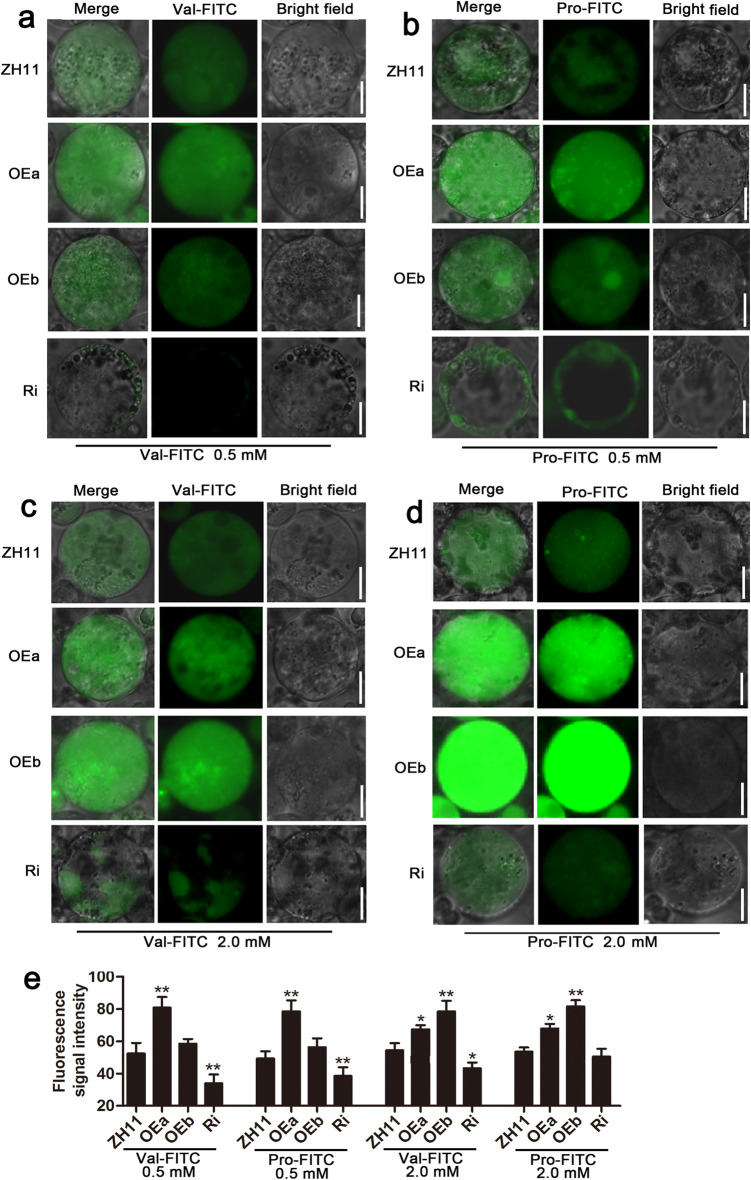
Protoplast amino acid-uptake assay among ZH11, OEa, OEb, and Ri lines. Fluorescence was detected after culturing protoplasts with FITC-labeled amino acids for 4 h. Green fluorescence images of ZH11 and OEa, OEb and Ri lines under treatment with 0.5 mM Val-FITC (**a**), 0.5 mM Pro-FITC (**b**), 2.0 mM Val-FITC (**c**), and 2.0 mM Pro-FITC (**d**). **e** Detection of cell fluorescence signal intensity in (**a**-**d**). Fluorescence intensities were normalized to the area of the respective cell by ImageJ software, and a total of 100 cells were statistically analyzed. Scale bars, 5.0 μm (**a**-**d**). The letters above the error bars are ranked by the T test, “*” indicates a significant difference at *p* < 0.05, and “**” indicates a significant difference at *p* < 0.01. Values are means ± s.d. (*n* = 3)

The protoplast-amino acid-FITC assay is a new method for examining plant amino acid transporters (Wang et al. 2019a; Ji et al. 2020). To further validate that OsAAP4 mediates Val and Pro transport, a protoplast amino acid-uptake assay was performed. Protoplasts were cultured with 0.5 mM and 2.0 mM fluorescein isothiocyanate (FITC)-labeled amino acids, Val-FITC and Pro-FITC. Stronger fluorescence signals in the cytoplasm were detected in the protoplasts of OEa lines cultured with 0.5 mM Val-FITC and 0.5 mM Pro-FITC for 4 h than those of the ZH11 and OEb lines, and the FITC signal was weaker in Ri lines than in ZH11 (Fig. 6a, b, e). Interestingly, when protoplasts were cultured with each FITC-labeled amino acid at 2.0 mM (Val-FITC, Pro-FITC) for 4 h, OEb lines presented stronger fluorescence signals than did ZH11 and OEa lines, and the opposite was found for Ri lines (Fig. 6c, d, e).

As the concentrations of amino acids Arg, Lys, Thr and Leu in *OsAAP4* transgenic plants also changed, protoplasts were cultured with Arg-FITC, Lys-FITC, Thr-FITC and Leu for amino acid transport of OsAAP4. We detected higher fluorescence signal intensity in the protoplasts of the Ri lines cultured with Lys-FITC and Arg-FITC than in ZH11 protoplasts, and the FITC signal was weaker in the OE lines than in ZH11 (Supplementary file 9: Figure S9a, b, e). However, higher fluorescence signal intensity in the protoplasts of the OE lines cultured with Thr-FITC and Leu-FITC than in ZH11 protoplasts, and FITC signal were lower in the Ri lines than in ZH11 (Supplementary file 9: Figure S9c, d, e). These results indicated that OsAAP4 might played a crucial role in regulating neutral amino acids in rice plant cells of different variants at different concentrations.

### *OsAAP4* Regulates Bud Outgrowth and Rice Tillering by Coordinating Nitrogen and Phytohormone Pathway

**Fig. 7:**
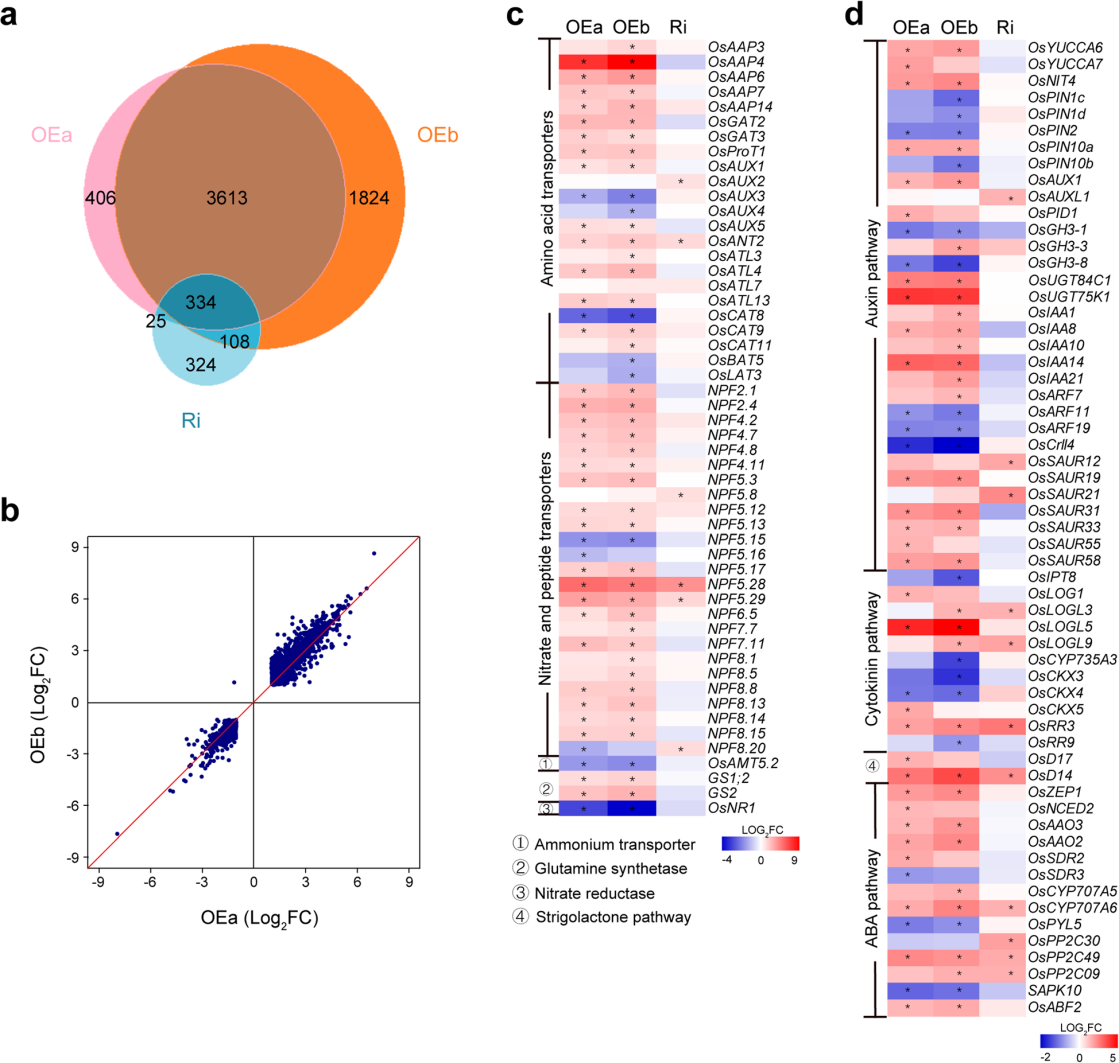
Transcriptome analysis of *OsAAP4* transgenic plants in the growing axillary buds. **a** Identification of differentially expressed genes (DEGs) in the axillary buds of OEa, OEb, and Ri lines of *OsAAP4* and wild-type ZH11 (adjusted *P*-value < 0.05 and fold change > 2). **b** Scatter plot of different genes compared OEa and ZH11, with OEb and ZH11. **c**-**d** Heatmap visualization of expression profiles of DEGs in nitrogen transport and metabolism (**c**), auxin, cytokinin, SL, and ABA signaling pathways (**d**). Red boxes show up-regulation, and green boxes show down-regulation

To investigate the mechanism of OsAAP4 in regulating bud outgrowth, we performed RNA-seq using RNA samples from the tiller buds of the *OsAAP4* OE lines, Ri lines and the wild-type ZH11. A total of 334 genes were differentially expressed between OEa, OEb and RNAi lines, and 3613 co-regulated downstream genes between OEa and OEb, however, OEb regulated more downstream genes (Fig. 7a). Scatter plot results showed that the gene patterns of OEa and OEb were very similar compared with ZH11 (Fig. 7b). To understand the biological functions of these Differentially Expressed Genes (DEGs), we performed Kyoto Encyclopedia of Genes and Genomes (KEGG) pathway enrichment analysis. The DEGs were assigned to one KEGG pathways (metabolic pathways) in *OsAAP4* OEa lines (Supplementary file 10: Figure S10a), 12 KEGG pathways, such as metabolic pathways, biosynthesis of secondary metabolites, Val, Leu and Ile degradation in *OsAAP4* OEb lines (Supplementary file 10: Figure S10b), 40 KEGG pathways, such as metabolic pathways, biosynthesis of secondary metabolites, Arg and Pro metabolism, Gly, Ser and Thr metebolism, and plant hormone signal transduction in both *OsAAP4* OEa and OEb lines (Supplementary file 11: Figure S11). To further investigate the mechanism of *OsAAP4* in regulating bud outgrowth, we analyzed the expression patterns of DEGs in N transport and metabolism, and the heatmap result showed that many amino acid transporters genes (such as *OsAAP4* and *OsAAP7*), nitrate and peptide transporters genes (such as *OsNPF2.4*, *OsNPF6.5*, and *OsNPF7.7*), glutamine synthetase genes (*OsGS1;2* and *OsGS2*) had increased expression in OEa or OEb lines, but reduced expression in Ri lines (Fig. 7c; Supplementary file 12: Figure S12), which indicated that altered expression of *OsAAP4* may influence the expression of other nitrogen transport genes and the glutamine synthetases needed for the regulation of the axillary bud outgrowth. In addition, the heatmap result showed that *YUCCA* auxin biosynthetic genes were up-regulated in the *OsAAP4* OE lines compared with the wild-type ZH11 (Fig. 7c), indicating that auxin may be decreased in the axillary buds of *OsAAP4* OE lines, leading to the down-regulation of the auxin transporter *PIN* genes (Fig. 7c), and resulting in the induction of the axillary bud outgrowth (Fig. 7c). Besides, the decreased expression of *OsCKX3* and *OsCKX4* may promote the cytokinin signaling, leading to the promotion of axillary bud outgrowth of *OsAAP4* OE lines (Fig. 7c). Moreover, the expression of the ABA biosynthesis and signaling genes was decreased to promote the bud outgrowth of *OsAAP4* OE lines (Fig. 7c). In order to further determine whether there is a regulatory relationship between *OsAAP4* and *OsAAP3* or *OsAAP5* that affect rice tillering, we detected the expression of *OsAAP4* in *OsAAP3* and *OsAAP5* transgenic plants. The result showed that the expression of *OsAAP4* in basal part of *OsAAP5* OE lines was higher, however, there was no consistent expression pattern in other transgenic plants (Supplementary file 13: Figure S13), suggesting that there is no direct relationship between *OsAAP4* and *OsAAP3* or *OsAAP5* in rice tillering regulation. These results indicated that altered expression of *OsAAP4* influenced bud outgrowth and rice tillering by coordinating nitrogen and phytohormone pathway.

## Discussion

Here, we provide evidence to support the hypothesis that the amino acid transporter OsAAP4 contributes to rice tillering and grain yield by regulating neutral amino acid transport through two different splicing variants. First, we found that the expression level of *OsAAP4* was higher in *Indica* than in *Japonica* and that upregulation of *OsAAP4* in *Japonica* significantly increased tiller number, grain yield and NUtE. However, *OsAAP3* and *OsAAP5* of the rice amino acid transporter family, are highly expressed in *Japonica* rice (Lu et al. 2018; Wang et al. 2019a), and blocking *OsAAP3* and *OsAAP5* in *Japonica* rice enhances tiller number and grain yield (Lu et al. 2018; Wang et al. 2019a). Previous studies have also demonstrated that the T-DNA insertion line *ataap2* exhibits strongly increased branch and silique numbers per plant as well as seed yield (Zhang et al. 2010). In contrast, overexpression of *PtAAP1* improves plant NUtE through alteration of amino acid transport from source-to-sink in pea (Perchlik and Tegeder, 2017). In rice, another organic nitrogen transporter of the NPF family, OsNPF7.3, transports di/tripeptides Gly-His and Gly-His-Gly (Ouyang et al. 2010) and positively influences rice tiller number and NUtE (Fang et al. 2017). A recent study reveals that the amino acid transporter OsAAP1 mediates growth and grain yield by regulating neutral amino acid uptake and reallocation in rice (Ji et al. 2020). Our study further indicated that as a result of artificial selection, different rice accessions are able to adapt to the environment by regulating expression of different AATs.

Second, the altered expression of *OsAAP4* relatively influenced the contents of amino acids Val and Pro both in rice seedling and reproductive stage, and the elongation of tiller bud of *OsAAP4* transgenic lines could also be regulated by exogenous amino acids Val and Pro, although the amino acid contents determined by HPLC can not distinguish the amino acids that were transported by OsAAP4 or altered indigenous synthesis. Furtermore, whether OsAAP4 can regulate the transport of these amino acids was verified using a new method of amino acid-FITC labeling and a protoplast uptake assay (Wang et al. 2019a; Ji et al. 2020), and we determined that both variants of *OsAAP4* could regulate neutral amino acid transport in rice plant cells. Furthermore, the longer variant OsAAP4a regulated Val and Pro transport at low concentrations, whereas the shorter variant OsAAP4b regulated Val and Pro transport at high concentrations. In *Arabidopsis*, there is only one variant of AtAAP4*,* which transports Val and Pro (Fischer et al. 1995), though this protein grouped into different subclusters compared with rice OsAAP4. Recent insight into the origin and evolution of AAP proteins has revealed that AAP proteins are mainly found in land vascular plants and that algae lack AAPs (Tegeder and Ward, 2012). The divergence of *AAP4* between rice and *Arabidopsis* indicates that different variants may play key roles in adapting to different soil nutritional conditions which encountered by rice in artificial cultivation. Similarly, there are two variants (OsNRT2.3a and OsNRT2.3b) of the rice high-affinity nitrate transporter OsNRT2.3, and OsNRT2.3b can sense pH changes in cells, thus facilitating the absorption of more nitrogen, iron and other nutrients (Fan et al. 2016). Overexpression of *OsNRT2.3b* might improve rice yield and NUtE (Fan et al. 2016). Recently, it was suggested that two splicing variants of OsNPF7.7 regulate tiller number and NUtE in rice, with OsNPF7.7a facilitating nitrate influx and concentration and OsNPF7.7b improving ammonium influx (Huang et al. 2018). Excitingly, our results indicate that two OE lines of *OsAAP4* promote rice growth under different Val and Pro concentrations.

Additionally, the neutral amino acid Val is an important branched-chain amino acid, and disruptions in Val degradation affect seed development and germination in *Arabidopsis* (Gipson et al. 2017). Our study showed that Val promoted growth in rice plants, especially bud outgrowth for tillers (Fig. 5). Another neutral amino acid, Pro, is critical for rapid cell division in organ development (Venekamp and Koot 1984; Lehmann et al. 2010), because rapidly dividing and growing cells have a high demand for Pro (Székely et al. 2008). PtAAP11, the plant amino acid transporter with the highest affinity for Pro, is mainly expressed in shoot and root meristematic cells and facilitates bud development (Couturier et al. 2010). In our study, treatment with moderate Val and Pro concentrations promoted plant height, biomass, and bud outgrowth in two OE lines, consistent with the finding that exogenously applied Pro improved the in vitro shoot regeneration frequency of rice (Pawar et al. 2015).

In addition, Pro plays a role as a compatible solute under environmental stress conditions (Lehmann et al. 2010). The Glu pathway is the primary route for Pro synthesis in plants during conditions of osmotic stress and nitrogen limitation, whereas the ornithine pathway assumes prominence under high nitrogen input (Delauney et al. 1993). Therefore, AAP4a may divert Pro from the Glu synthesis pathway when nitrogen is limited, whereas AAP4b may acquire Pro from the ornithine synthesis pathway when nitrogen is abundant. Lys can inhibit mitotic activity in the root apical meristem, and higher exogenous Lys can reduce the length of the main root of *Arabidopsis* (Yang et al. 2014) and inhibit bud outgrowth in rice (Lu et al. 2018; Wang et al. 2019a). *OsAAP4* RNAi both reduced the concentration of neutral amino acids (Val and Pro) and increased that of basic amino acids (Lys and Arg), which may explain why Ri lines exhibited worse growth than wild-type ZH11. Downregulation of *OsAAP4* affected bud outgrowth, plant height, and biomass by regulating neutral amino acids (Val and Pro) and basic amino acids (Lys and Arg) in rice.

Finally, tiller number is an important feature of the rice grain yield produced from bud initiation and elongation (Li et al. 2003), and tiller bud outgrowth is regulated by both environmental signals and endogenous factors (Xing and Zhang 2010; Fang et al. 2020). Of all the nitrogen transporters characterized to date, only *OsNPF8.20*, *OsNPF6.5*, *OsNPF7.3*, *OsNPF7.2*, *OsNPF7.7*, and *OsAAP1 *can positively regulate rice tiller number and enhance grain yield (Fang et al. 2013; Hu et al. 2015; Fang et al. 2017; Wang et al. 2018; Huang et al. 2018; Ji et al. 2020). Our results indicated that overexpression of *OsAAP4* also positively influences tiller number by regulating expression of *OsNPF6.5* and *OsNPF7.7* (Fig. 7c). Recently, the genes *OsGS1;2* and *OsGS2* were found to be highly expressed in the axillary buds under 5.0 mM nitrogen (Wang et al. 2020), and further indicated that overexpression of *OsGS1;2* and *OsGS2* promote axillary bud growth and tiller number via ammonium assimilation, whereas reduced expression of *GS1;2* affects the assimilation of ammonium into glutamine, resulting in decreased bud elongation and tiller number in rice (Ohashi et al. 2015; Wang et al. 2020). Similarly, our experiment also showed that expression of two genes *OsGS1;2* and *OsGS2* of the nitrogen pathway was increased in OE lines but decreased in Ri lines of *OsAAP4*. Taken together, these results demonstrate that altered expression of *OsAAP4* influences bud outgrowth through the nitrogen and phytohormone pathway. It has been reported that the phytohormone cytokinin (CK) promotes tillering (Dun et al., 2012), while auxin can inhibit tillering (Leyser, 2003). Our study indicated that the expression of such CK crucial genes as *OsCKX3* and *OsCKX4* was lower in OE lines than in ZH11 (Fig. 7d), suggesting that CKs probably produced in larger amounts in OE lines than in ZH11. Moreover, the expression of *OsYUCCA6*, *OsYUCCA7* was higher in OE lines than in ZH11, whereas the expression of *OsPIN1c*, *OsPIN1d*, *OsPIN2*, and *OsPIN10b* was lower in OE lines than in ZH11 (Fig. 7c), indicating that auxin may be decreased in the axillary buds of *OsAAP4* OE lines, resulting in the induction of the axillary bud outgrowth.

### Conclusions

In this study, we demonstrate that *OsAAP4* promoter sequences are divergent between *Indica* and *Japonica*, and overexpression of two different splicing variants of *OsAAP4* in *Japonica* ZH11 significantly promoted rice tillering and grain yield as result of enhancing the neutral amino acid concentrations. Importantly, *OsAAP4* positively regulated tiller bud outgrowth probably by coordinating nitrogen transport and metabolism, and auxin, CK signaling pathway.

## Methods

### Plasmid Construction

To construct an *OsAAP4a* or *OsAAP4b-*overexpression plasmid, a 1407-bp fragment of *OsAAP4a* cDNA or a 1116-bp fragment of *OsAAP4b* cDNA containing the open reading frame (ORF) was inserted downstream of the *35S* promoter of the pCAM1306 vector digested using *Kpn*I and *XbaI*, respectively, to produce *p35S*-*OsAAP4a* and *p35S*-*OsAAP4b*. To construct the *OsAAP4*-RNAi plasmid, two fragments of *OsAAP4* cDNA (263 bp) were amplified by PCR and cloned downstream of the *Ubi-1* promoter in the rice RNAi vector pTCK303 and digested by *BamH*I/*Kpn*I and *Spe*I/*Sac*I, respectively*.* The *OsAAP4* CRISPR plasmid was constructed using CRISPR/Cas9-based multiplex genome editing for monocot and dicot plants (Ma et al. 2015). To construct the *OsAAP4* promoter-GUS plasmid, a sequence of approximately 2500 bp upstream of the first ATG of *OsAAP4* in *Indica* W144 was inserted upstream of the *GUS* gene in pCAM1391Z using *Hind*III and *Nco*I to produce *pW144-GUS*, respectively. All primers used in this study are listed in Supplementary file 14: Table S1.

#### Plant Materials

*Japonica* Zhonghua 11 (ZH11) was transformed using *Agrobacterium*-mediated transformation, and transgenic calli were selected using 50 mg L^− 1^ hygromycin. T_2_ homologous transgenic lines were used in all experiments. All transgenic plants and 497 sequencing accessions (Chen et al. 2014) were grown at the rice experimental base in Wuhan and Sanya of Huazhong Agricultural University, China. Tiller number and other agronomic traits were measured at the filling stage over three seasons from 2014 to 2018. In general, 30 rice plants were used for each experiment, and the planting density was 19.98 cm × 19.98 cm.

#### RNA Extraction and PCR Analysis

Total RNA was extracted using TRIzol reagent according to the manufacturer’s instructions (TAKARA). First-strand cDNA was synthesized from 3 μg of total RNA treated with DNase I using M-MLV reverse transcriptase (TAKARA). The first-strand cDNA was used as the template for real-time quantitative PCR (RT-PCR) using normalization to rice Actin1 (LOC_Os03g50885). RT-PCR was performed in a 20 μL reaction volume containing 1 μL of cDNA solution, 1 × PCR buffer, 0.25 μM dNTPs, 1.0 μM gene-specific primers and 0.5 U of Taq polymerase (Takara) with the following conditions: 94 °C for 2 min (1 cycle); 94 °C for 30 s, 55 °C for 30 s, and 72 °C for 30 s (40 cycles); and 72 °C for 1 min (1 cycle). Amplification of the cDNA or promoter sequence of *OsAAP4* was performed in a 20 μL reaction volume containing 1 μL of cDNA or DNA solution, 1 × PCR buffer, 0.5 μM dNTPs, 1.0 μM gene-specific primers and 0.5 U of Taq polymerase (Takara) with the following conditions: 94 °C for 3 min (1 cycle); 94 °C for 30 s, 48–65 °C for 30 s, and 72 °C for 2 min (30–40 cycles); and 72 °C for 10 min (1 cycle).

#### Amino Acid and Total Nitrogen Analyses

Total and single free amino acid concentrations were measured by HPLC with an amino acid analyzer L-8800 HITACHI. The samples were prepared as follows. Rice tissue (1 g) was placed in 80% ethanol (10 ml) at 80 °C in a water bath for 20 min; this step was repeated twice. The collected extracts were placed at 80 °C in a drying oven to remove the ethanol, and the sediment was dissolved in 1 ml 0.5 M NaOH. The solution was centrifuged at 14,000 rpm for 15 min. The supernatant was collected and filtered through a filter membrane (2 μm); 0.8 ml of each filtrate was analyzed using an amino acid analyzer. The total nitrogen content and total protein content were determined using the semi-micro Kjeldahl method with a nitrogen analyzer (Smart Chem 200). Nitrogen utilization efficiency was determined using the formula: NUtE (%) = [grain yield (g) / (grain nitrogen content (g) + straw nitrogen content (g)] × 100.

#### GUS Staining

GUS staining of *pW144-GUS* of *OsAAP4* promoter-GUS transgenic plants was performed as described previously (Fang et al. 2017). All samples for GUS staining were vacuum infiltrated for 15 min and gently fixed in FAA (formalin-acetic acid-70% ethanol [1:1:18]) at 4 °C for 20–30 min. The samples were then incubated in staining buffer at 37 °C overnight. After removing chlorophyll by incubation in a solution of 80% ethanol, the stained samples were observed using a stereomicroscope OLYMPUS SZX16. Finally, the samples were embedded in Spurr resin and sectioned. The sections were observed using a Zeiss Axio Imager M2.

#### Hydroponic Culture and Plant Growth Observation

Transgenic *OsAAP4* plants were cultured in basic nutrient solution (Yoshida, 1976) with 1.0 mM NH_4_NO_3_ under natural rice growth conditions, and individual amino acids were adjusted in each experiment. To investigate the effect of neutral amino acids Val and Pro on the phenotype of *OsAAP4*-transgenic plants, seedlings were grown in basic rice culture solution with 1.0 mM NH_4_NO_3_ as the N source for 1 week and transferred to basic rice culture solution supplemented with 1.0 mM NH_4_NO_3_ and each amino acid as the N source. To assess axillary bud outgrowth, the first and second bud lengths of axillary buds were measured using a stereomicroscope and ImageJ software from 28 days after sowing. For hydroponic culture, different transgenic seedlings were grown in boxes (525 mm × 360 mm × 230 mm) in rice culture solution under greenhouse conditions of 32 °C with a sodium lamp at 400 W for 14 h (daytime) and 25 °C for 10 h (nighttime). The nutrient solution was renewed every 3 days.

#### Protoplast Amino Acid Uptake Assay

Amino acids labeled with FITC (Val-FITC, Pro-FITC, Thr-FITC and Leu-FITC, Arg-FITC, Lys-FITC) were synthesized by Yuan Peptide Biotechnology Company, Nanjing, China, as fluorescently tagged GA_3_-FITC in elongating endodermal cells of roots in *Arabidopsis* (Tal et al. 2016), and a protoplast biomolecule uptake assay was performed as previously described (Rottmann et al. 2018; Ji et al. 2020). Rice protoplasts prepared from etiolated seedlings of ZH11 and transgenic lines were incubated in 1 ml W5 buffer (pH 5.6) with each FITC-labeled amino acid at room temperature in the dark. Four hours later, the protoplasts were washed eight times to remove free amino acids, and fluorescence was observed using a confocal laser scanning microscope (Leica SP8). Rice protoplast cells with fluorescent amino acids were found at 10 × 100 times under the excitation light with a wavelength of 488 nm. The fluorescence intensity was modulated by 20%, and the photo resolution was 2048 × 2048.

#### Subcellular Localization

For subcellular localization of two variants of *OsAAP4*, *OsAAP4a* or *OsAAP4b*, the ORF was amplified and fused with green fluorescent protein (GFP) in the pCAM1302 vector to generate the p35S:*OsAAP4a*-*GFP* and p35S:*OsAAP4b*-*GFP* plasmid. The plasmid was transiently expressed in rice protoplasts prepared from etiolated seedlings of ZH11, and fluorescence was observed using a confocal laser scanning microscope (Leica SP8). The *Agrobacterium* strain GV3101 transformed with p35S:*OsAAP4a*-*GFP* and p35S:*OsAAP4b*-*GFP* was infiltrated into 1-month-old *N. benthamiana* plants to study the transient expression of *OsAAP4*. The DAPI, GFP, and FM4–64 fluorescence signals were detected at 2 d post-injection using a confocal laser scanning microscope (Leica SP8).

#### RNA-Seq Analysis

The axillary buds from transgenic *OsAAP4* plants and the wild-type ZH11 plants were collected for RNA sequencing (RNA-seq), analysis and two biological replicates were performed for each sample by Novogene. The clean data were aligned to the rice genome reference sequence (*Oryza_sativa*. IRGSP-1.0) by HiSAT2 (v2.1.0) (Kim et al. 2015). Transcripts were then assembled by stringtie (v2.0.1) (Pertea et al. 2016) and then processed by feature Counts to summarize the counting reads (subread-2.0.0) (Liao et al. 2014). The intersection of differential genes analyzed by DESeq2 [false discovery rate (FDR) < 0.05 and fold change≥2] were identified as differentially expressed genes (DEGs) (Love et al. 2014).

#### Statistical Analysis

Differences were analyzed using Student’s t and Duncan test, with the following significance levels: ***P < 0.001; **P < 0.01; * *P* < 0.05 or letters at *P* < 0.05.

## Supplementary Information

**Additional file 1: Figure S1.** Expression levels of *OsAAP4* in young seedling root (**a**), basal part (**b**), old leaf (**c**), young leaf (**d**) between Hap2 and Hap5 of five individual varieties. The primers used for quantifying *OsAAP4* expression was F: GACATCGTCCACAACCTCAAGGCT, and R: GCCACAGCTCTAGCTAGGCAGC. Values are means ± s.d. (*n*=3).

**Additional file 2: Figure S2.** Sequencing of two different types of promoter sequences of *OsAAP4*. NIP indicates Nipponbare, C172 indicates the Hap5 type in *Japonica*. W144 indicates the Hap2 type in *Indica*.

**Additional file 3: Figure S3.** Expression levels of *OsAAP4a* (**a**) and *OsAAP4b* (**b**) in young seedling basal part between Hap2 and Hap5 of five individual varieties. The primers used for quantifying *OsAAP4a* expression was F: TGGCACTCACCCTTGCACAC, and R: CCGTCCACACCGTCCCTTGT, for quantifying *OsAAP4b* expression was ACTTGAGCTCTCTGCATTGGGT, and R: AGCGGTAGCAATTGGCGAGGA. Values are means ± s.d. (*n*=3).

**Additional file 4: Figure S4.** Subcellular localization of OsAAP4*.* (**a**-**e**) Localization of the 35S promoter-driven GFP as the control in rice protoplasts. (**f**-**j**) Localization of the OsAAP4a-GFP in rice protoplasts. (**k**-**o**) Localization of the OsAAP4b-GFP in rice protoplasts. (**p**-**s**) Localization of OsAAP4a-GFP in tobacco pavement cells. (**t**-**w**) Localization of OsAAP4b-GFP in tobacco pavement cells. Green, GFP signal. Blue, DAPI (a nuclear marker) signal. Red, FM4-64 (a lipophilic membrane marker) signal. DIC, bright field. Scale bars represent 5 μm in (**a**-**o**) and 25 μm (**p**-**w**).

**Additional file 5: Figure S5.** Knockout of *OsAAP4* significantly decreased NUtE in rice *Japonica* ZH11 using CRISPR technology. **a** Sequencing results of the base addition of *OsAAP4*-CRISPR in *Japonica* ZH11 with CRISPR technology. Whole-plant phenotype (**b**) and grain yield per plant (**c**) of ZH11 and *OsAAP4-*CRISPR lines in the ZH11 background. Quantification of tiller number per plant (**d**), filled grain yield per plant (**e**), grain yield per plant (**f**), and NUtE (**g**) of ZH11 and *OsAAP4-*CRISPR lines. The letters above the error bars are ranked by the T test, “***” indicates a significant difference at *p*<0.001. Scale bars, 5.0 cm (**b**), 3.0 cm (**c**). Values are means ± s.d. (*n*>20).

**Additional file 6: Figure S6**. Phenotypic analysis of *OsAAP4* transgenic plants in the *Japonica* ZH11 background grown in Sanya paddy fields. (**a**) Whole-plant phenotype. (**b**) Tiller number per plant. OEa-1 and OEa-2 indicate long variants of *OsAAP4a-*overexpressing lines, OEb-1 and OEb-2 indicate short variants *OsAAP4b-*overexpressing lines, Ri-1 and Ri-2 indicate *OsAAP4*-RNAi lines, and *osaap4* indicates *OsAAP4-*CRISPR line. The letters above the error bars are ranked by the T test, “*” indicates a significant difference at *p*<0.05, and “***” indicates a significant difference at *p*<0.001. Scale bar, 10.0 cm (**a**). Values are means ± s.d. (n>20).

**Additional file 7: Figure S7.** Total free amino acid concentration of basal parts at seedlings stage and straw at filling stage. OEa, OEb, and Ri indicated that mixed equal-amount which extracted from each three OEa, OEb, and Ri lines, respectively. The letters above the error bars are ranked by the T test, “*” indicates a significant difference at *p*<0.05, and “**” indicates a significant difference at *p*<0.01. Values are means ± s.d. (*n*=3).

**Additional file 8: Figure S8.** Effect of different concentrations of Val and Pro on the growth of ZH11, OEa, OEb, and Ri lines grown in hydroponic culture. Phenotypes of seedlings among ZH11, OEa, OEb, and Ri lines grown with 1.0 mM NH_4_NO_3_ and Val 0.5 mM (**a**), Val 2.0 mM (**b**), Pro 0.5 mM (**c**), and Pro 2.0 mM (**d**). Quantification of plant height (**e**) and biomass (**f**) under Val 0.5 mM and Val 2.0 mM treatment. Quantification of plant height (**g**) and biomass (**h**) under Pro 0.5 mM and Pro 2.0 mM treatment. The letters above the error bars are ranked by the T test, “*” indicates a significant difference at *p*<0.05, “**” indicates a significant difference at *p*<0.01, and “***” indicates a significant difference at *p*<0.001. Scale bar, 10.0 cm (a-d). Values are means ± s.d. (*n*>15).

**Additional file 9: Figure S9.** Protoplast amino acid-uptake assay among ZH11, OEa, OEb, and Ri lines. Fluorescence was detected after culturing protoplasts with FITC-labeled amino acids for 4 h. Green fluorescence images of ZH11 and OEa, OEb and Ri lines under treatment with 0.5 mM Arg-FITC (**a**), 0.5 mM Lys-FITC (**b**), 0.5 mM Thr-FITC (**c**), and 0.5 mM Leu-FITC (**d**). **e** Detection of cell fluorescence signal intensity in (**a**-**d**). Fluorescence intensities were normalized to the area of the respective cell by ImageJ software, and a total of 100 cells were statistically analyzed. Scale bars, 5.0 μm (**a**-**d**). The letters above the error bars are ranked by the T test, “*” indicates a significant difference at *p*<0.05, “**” indicates a significant difference at *p*<0.01, and “***” indicates a significant difference at *p*<0.001. Values are means ± s.d. (n=3).

**Additional file 10: Figure S10.** KEGG enrichment analysis of the DEGs in the axillary buds of genes that are respectively regulated by OEa lines (**a**), OEb lines (**b**) compared with the wild-type ZH11. Gene ratio indicates that the ratio of the DEG number and the number of genes has been annotated in this pathway.

**Additional file 11: Figure S11.** KEGG enrichment analysis of the DEGs in the axillary buds of genes that are jointly regulated by OEa and OEb lines compared with the wild-type ZH11. Gene ratio indicates that the ratio of the DEG number and the number of genes has been annotated in this pathway.

**Additional file 12: Figure S12**. Heatmap visualization of expression profiles of DEGs in OsAAPs regulated by *OsAAP4* OEa and OEb lines compared with the wild-type ZH11. Red boxes show up-regulation, and green boxes show down-regulation. “*” indicates a significant difference at *P*-value <0.05 and fold change >2.

**Additional file 13: Figure S13.** The expression of *OsAAP4* in basal part of *OsAAP3* and *OsAAP5* transgenic plants. The primers used for quantifying *OsAAP4* expression was F: GACATCGTCCACAACCTCAAGGCT, and R: GCCACAGCTCTAGCTAGGCAGC. The letters above the error bars are ranked by the T test, “***” indicates a significant difference at *p* < 0.001. Values are means ±SD (n = 3).

**Additional file 14 Table S1.** List of the primers in this study.

## Data Availability

All data supporting the conclusions of this article are provided within the article (and its additional files).

## Abbreviations

AAP: Amino acid permease
AAT: Amino acid transporter
Arg: Arginine
Asp: Aspartic acid
CK: Cytokinin
CRISPR: Clustered regularly interspaced short palindromic repeats
Cys: Cysteine
DEG: Differentially expressed gene
FDR: False discovery rate
FITC: Fluorescein isothiocyanate
GFP: Green fluorescent protein
Glu: Glutamic acid
Gly: Glycine
GUS: β-glucuronidase
Haplotype: Hap
HPLC: High performance liquid chromatography
Ile: Isoleucine
KEGG: Kyoto encyclopedia of genes and genomes
Leu: Leucine
Lys: Lysine
NutE: Nitrogen utilization efficiency
OE: Over-expression/Overexpresing
ORF: Open reading frame
Phe: Phenylalanine
Pro: Proline
Ri: RNA interference
RNA-seq: RNA sequencing
RT-PCR: Real-time quantitative PCR
Ser: Serine
SNP: Single-nucleotide polymorphisms
Thr: Threonine
Tyr: Tyrosine
Val: Valine
ZH11: Zhonghua 11
