# Supplementary figures and images for: The Amino Acid Transporter OsAAP4 Contributes to Rice Tillering and Grain Yield by Regulating Neutral Amino Acid Allocation through Two Splicing Variants
Source: Rice (N Y). 2021 Jan 6;14:2. doi: 10.1186/s12284-020-00446-9 (PMC7788160; doi:10.1186/s12284-020-00446-9)

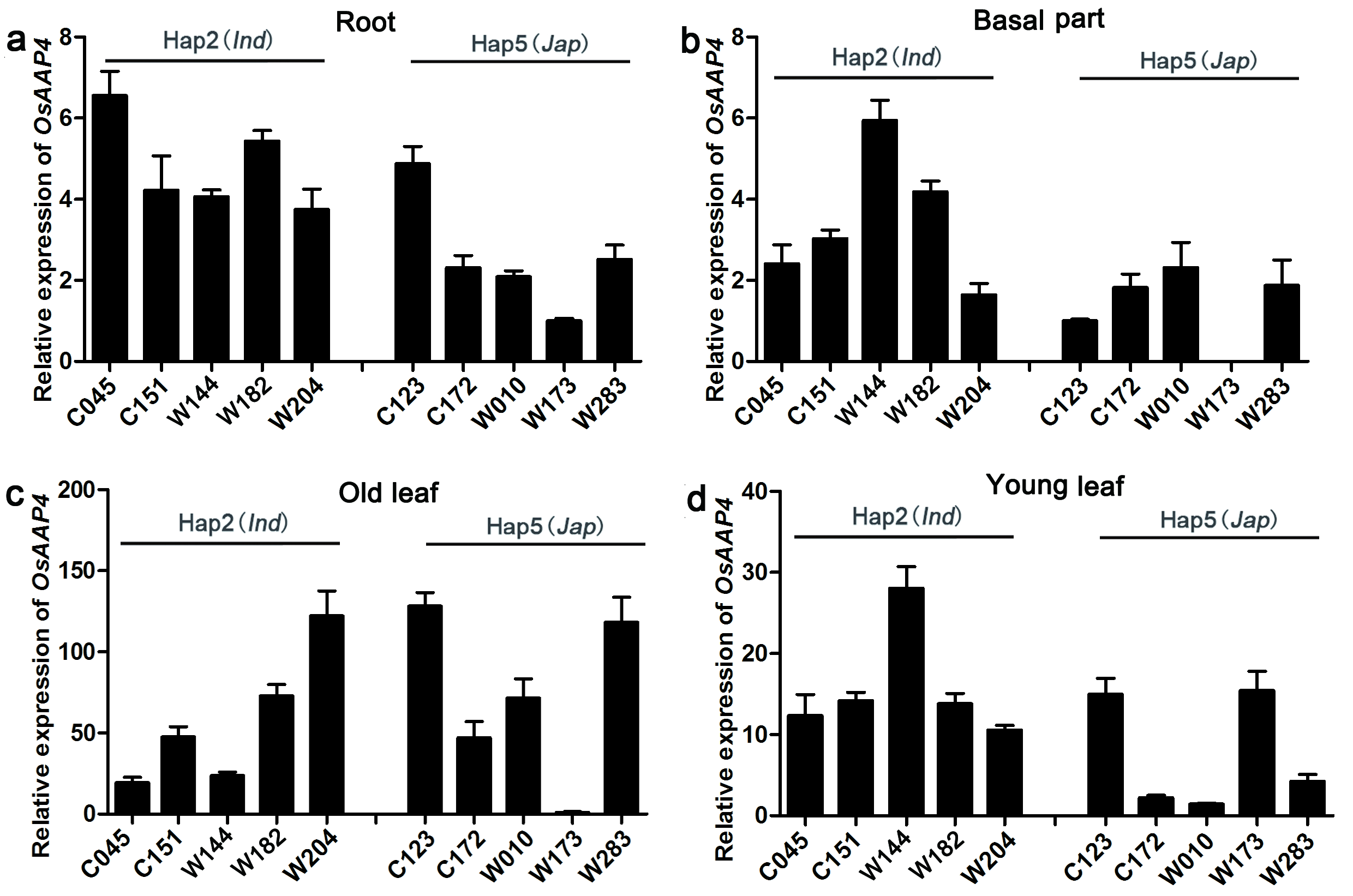

Supplement: Supplementary file 1 — Additional file 1: Figure S1. Expression levels of OsAAP4 in young seedling root (a), basal part (b), old leaf (c), young leaf (d) between Hap2 and Hap5 of five individual varieties. The primers used for quantifying OsAAP4 expression was F: GACATCGTCCACAACCTCAAGGCT, and R: GCCACAGCTCTAGCTAGGCAGC. Values are means ± s.d. (n=3). [file 12284_2020_446_MOESM1_ESM.tif]

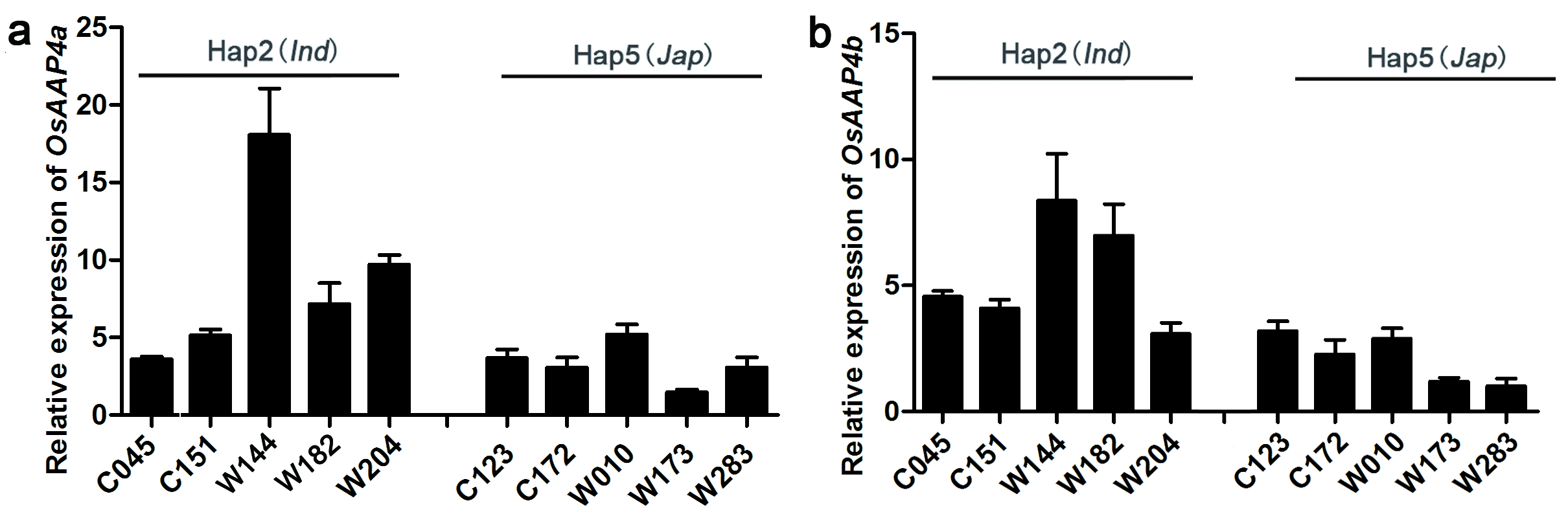

Supplement: Supplementary file 3 — Additional file 3: Figure S3. Expression levels of OsAAP4a (a) and OsAAP4b (b) in young seedling basal part between Hap2 and Hap5 of five individual varieties. The primers used for quantifying OsAAP4a expression was F: TGGCACTCACCCTTGCACAC, and R: CCGTCCACACCGTCCCTTGT, for quantifying OsAAP4b expression was ACTTGAGCTCTCTGCATTGGGT, and R: AGCGGTAGCAATTGGCGAGGA. Values are means ± s.d. (n=3). [file 12284_2020_446_MOESM3_ESM.tif]

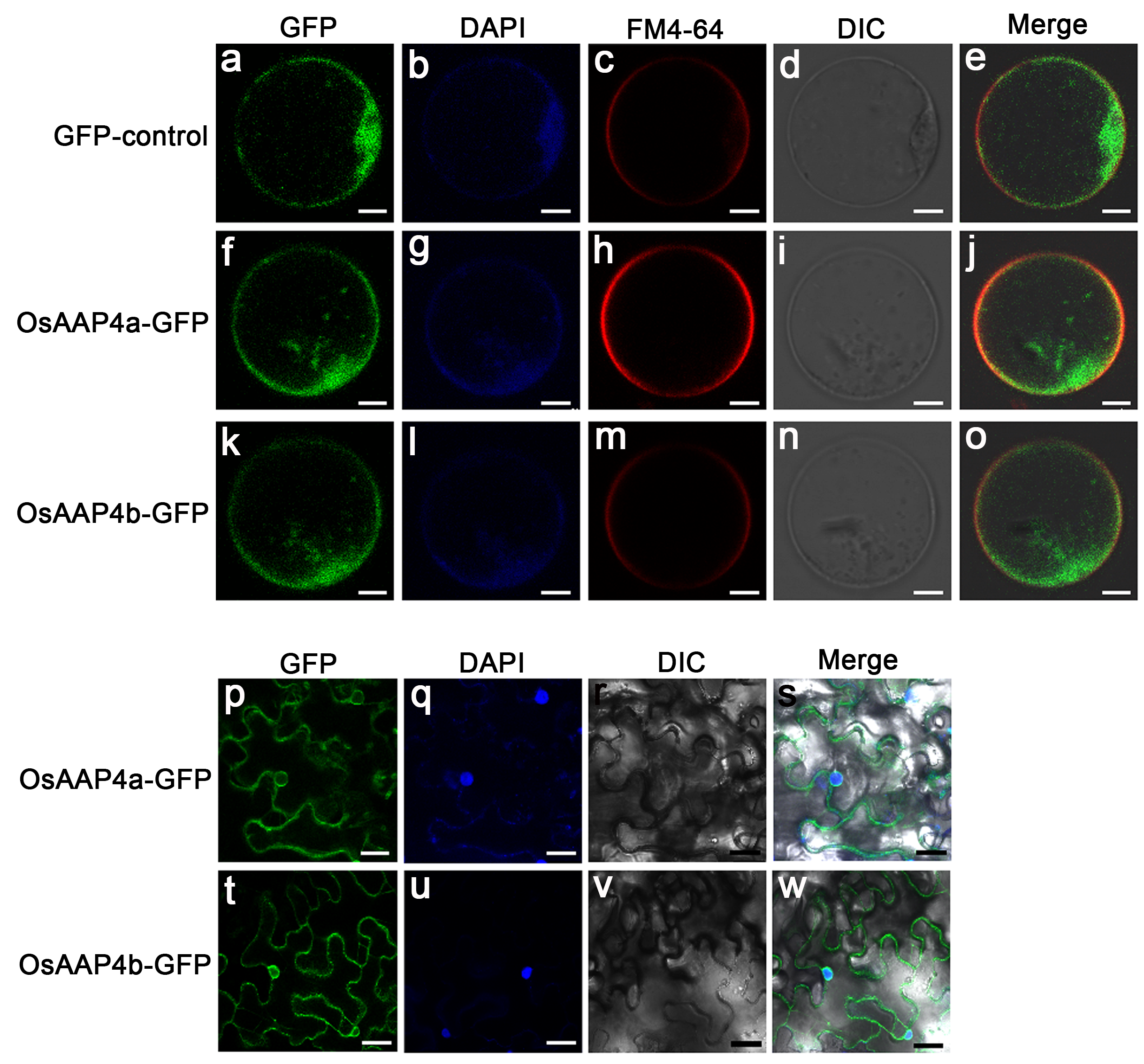

Supplement: Supplementary file 4 — Additional file 4: Figure S4. Subcellular localization of OsAAP4. (a-e) Localization of the 35S promoter-driven GFP as the control in rice protoplasts. (f-j) Localization of the OsAAP4a-GFP in rice protoplasts. (k-o) Localization of the OsAAP4b-GFP in rice protoplasts. (p-s) Localization of OsAAP4a-GFP in tobacco pavement cells. (t-w) Localization of OsAAP4b-GFP in tobacco pavement cells. Green, GFP signal. Blue, DAPI (a nuclear marker) signal. Red, FM4-64 (a lipophilic membrane marker) signal. DIC, bright field. Scale bars represent 5 μm in (a-o) and 25 μm (p-w). [file 12284_2020_446_MOESM4_ESM.tif]

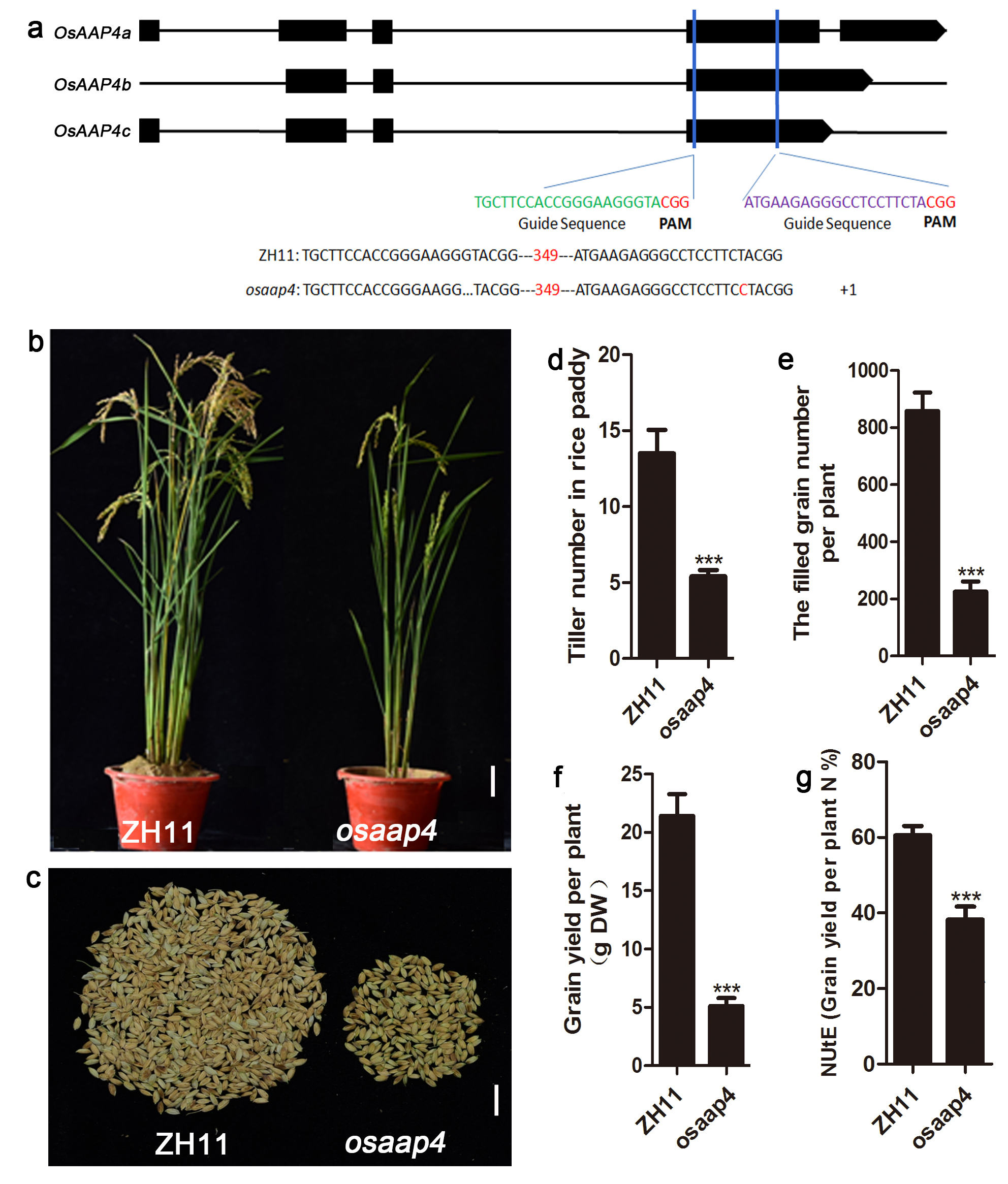

Supplement: Supplementary file 5 — Additional file 5: Figure S5. Knockout of OsAAP4 significantly decreased NUtE in rice Japonica ZH11 using CRISPR technology. a Sequencing results of the base addition of OsAAP4-CRISPR in Japonica ZH11 with CRISPR technology. Whole-plant phenotype (b) and grain yield per plant (c) of ZH11 and OsAAP4-CRISPR lines in the ZH11 background. Quantification of tiller number per plant (d), filled grain yield per plant (e), grain yield per plant (f), and NUtE (g) of ZH11 and OsAAP4-CRISPR lines. The letters above the error bars are ranked by the T test, “***” indicates a significant difference at p<0.001. Scale bars, 5.0 cm (b), 3.0 cm (c). Values are means ± s.d. (n>20). [file 12284_2020_446_MOESM5_ESM.tif]

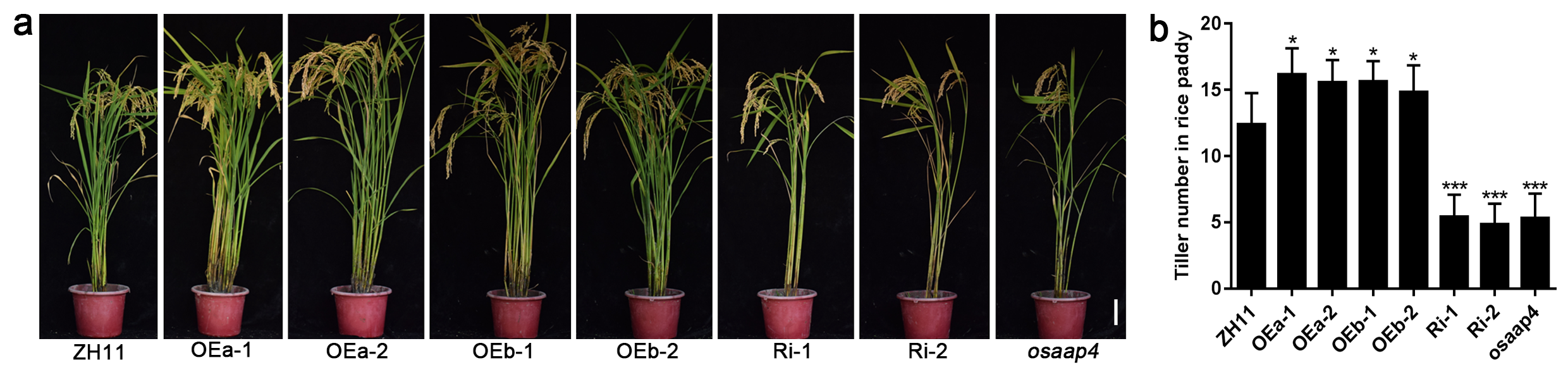

Supplement: Supplementary file 6 — Additional file 6: Figure S6. Phenotypic analysis of OsAAP4 transgenic plants in the Japonica ZH11 background grown in Sanya paddy fields. (a) Whole-plant phenotype. (b) Tiller number per plant. OEa-1 and OEa-2 indicate long variants of OsAAP4a-overexpressing lines, OEb-1 and OEb-2 indicate short variants OsAAP4b-overexpressing lines, Ri-1 and Ri-2 indicate OsAAP4-RNAi lines, and osaap4 indicates OsAAP4-CRISPR line. The letters above the error bars are ranked by the T test, “*” indicates a significant difference at p<0.05, and “***” indicates a significant difference at p<0.001. Scale bar, 10.0 cm (a). Values are means ± s.d. (n>20). [file 12284_2020_446_MOESM6_ESM.tif]

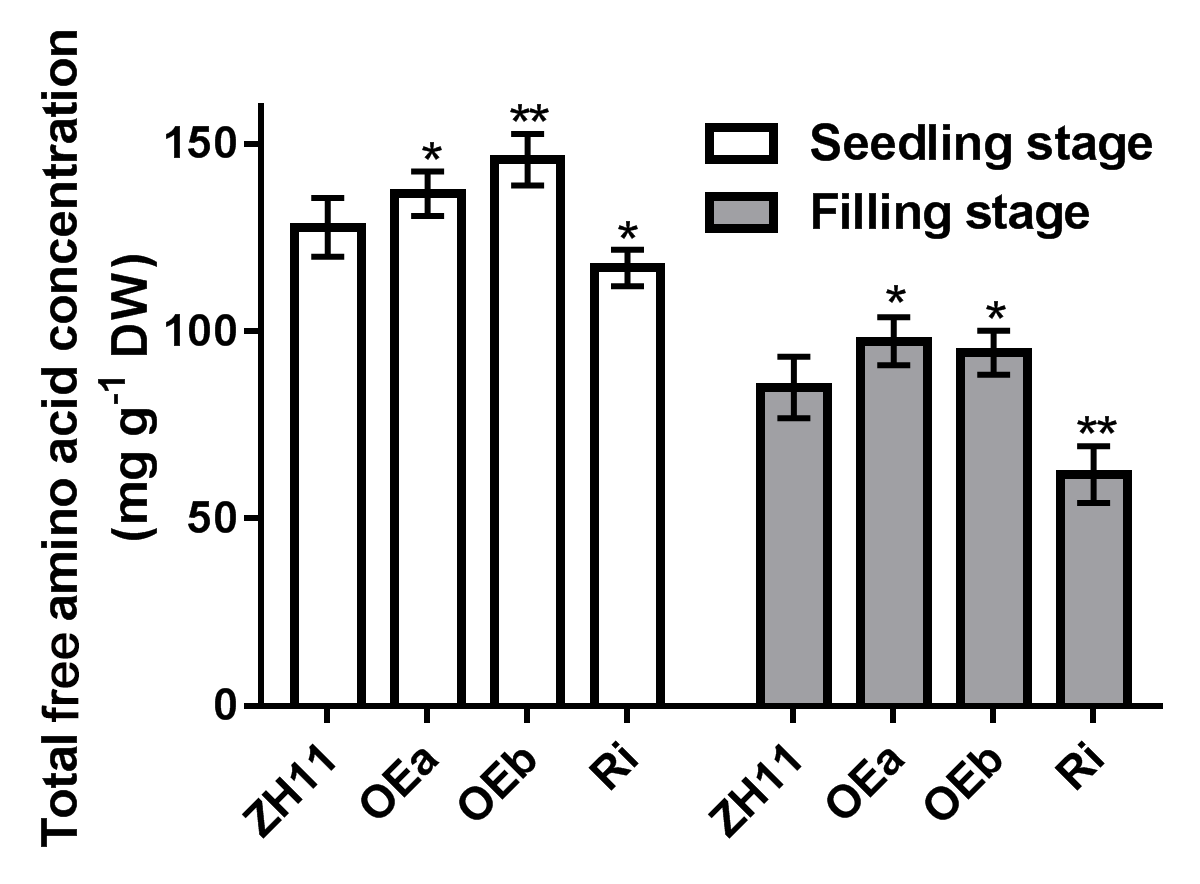

Supplement: Supplementary file 7 — Additional file 7: Figure S7. Total free amino acid concentration of basal parts at seedlings stage and straw at filling stage. OEa, OEb, and Ri indicated that mixed equal-amount which extracted from each three OEa, OEb, and Ri lines, respectively. The letters above the error bars are ranked by the T test, “*” indicates a significant difference at p<0.05, and “**” indicates a significant difference at p<0.01. Values are means ± s.d. (n=3). [file 12284_2020_446_MOESM7_ESM.tif]

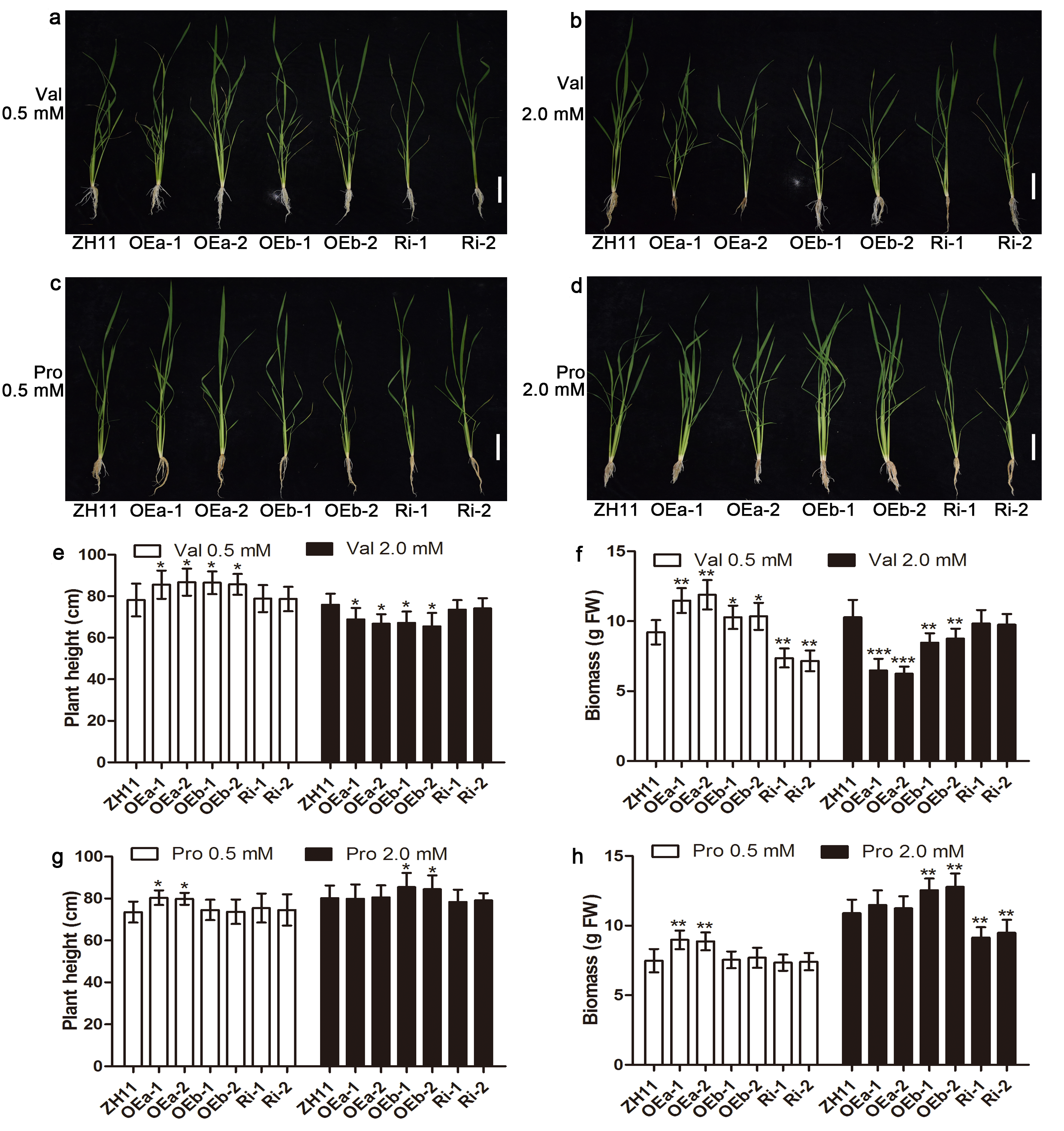

Supplement: Supplementary file 8 — Additional file 8: Figure S8. Effect of different concentrations of Val and Pro on the growth of ZH11, OEa, OEb, and Ri lines grown in hydroponic culture. Phenotypes of seedlings among ZH11, OEa, OEb, and Ri lines grown with 1.0 mM NH4NO3 and Val 0.5 mM (a), Val 2.0 mM (b), Pro 0.5 mM (c), and Pro 2.0 mM (d). Quantification of plant height (e) and biomass (f) under Val 0.5 mM and Val 2.0 mM treatment. Quantification of plant height (g) and biomass (h) under Pro 0.5 mM and Pro 2.0 mM treatment. The letters above the error bars are ranked by the T test, “*” indicates a significant difference at p<0.05, “**” indicates a significant difference at p<0.01, and “***” indicates a significant difference at p<0.001. Scale bar, 10.0 cm (a-d). Values are means ± s.d. (n>15). [file 12284_2020_446_MOESM8_ESM.tif]

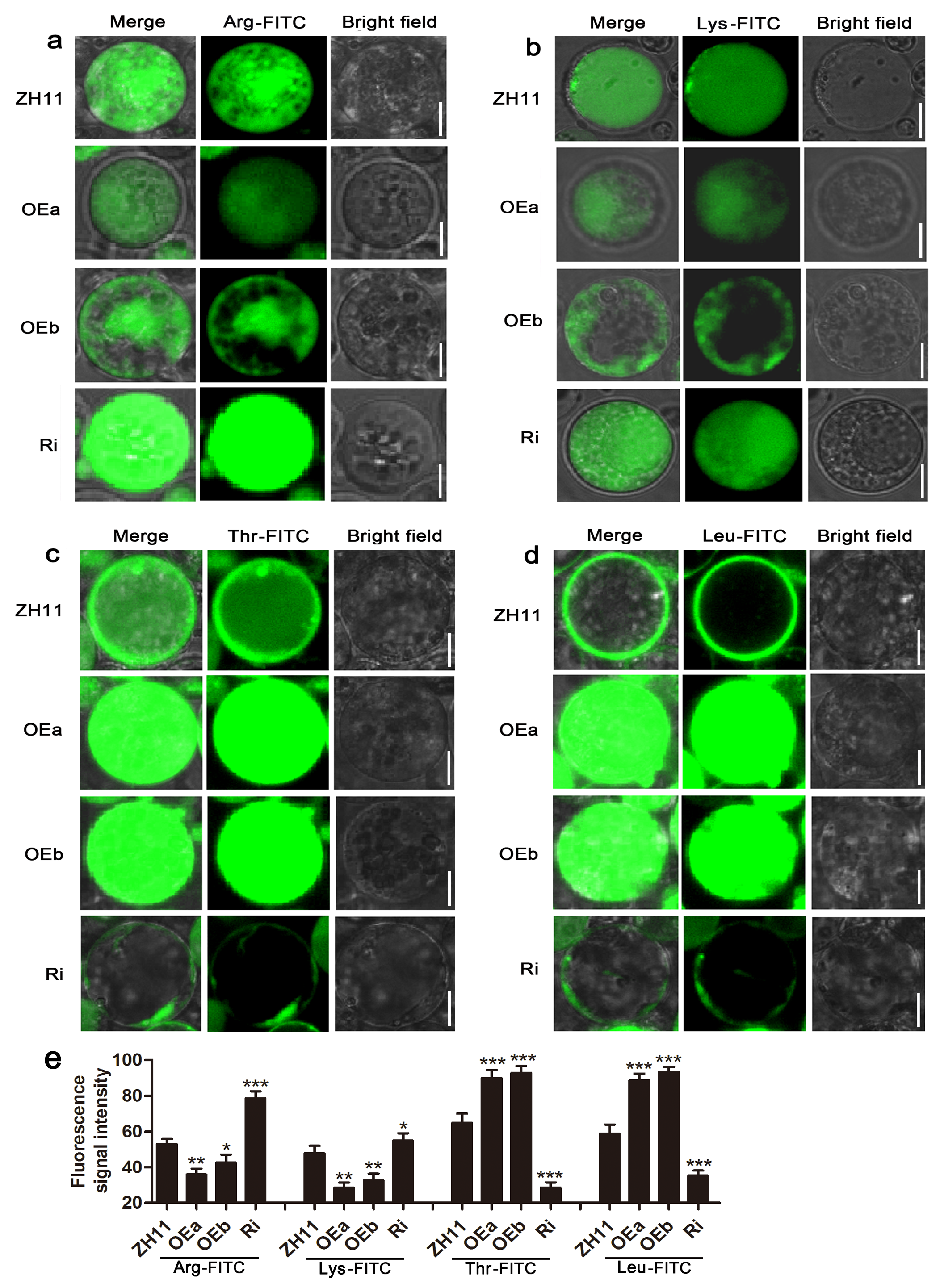

Supplement: Supplementary file 9 — Additional file 9: Figure S9. Protoplast amino acid-uptake assay among ZH11, OEa, OEb, and Ri lines. Fluorescence was detected after culturing protoplasts with FITC-labeled amino acids for 4 h. Green fluorescence images of ZH11 and OEa, OEb and Ri lines under treatment with 0.5 mM Arg-FITC (a), 0.5 mM Lys-FITC (b), 0.5 mM Thr-FITC (c), and 0.5 mM Leu-FITC (d). e Detection of cell fluorescence signal intensity in (a-d). Fluorescence intensities were normalized to the area of the respective cell by ImageJ software, and a total of 100 cells were statistically analyzed. Scale bars, 5.0 μm (a-d). The letters above the error bars are ranked by the T test, “*” indicates a significant difference at p<0.05, “**” indicates a significant difference at p<0.01, and “***” indicates a significant difference at p<0.001. Values are means ± s.d. (n=3). [file 12284_2020_446_MOESM9_ESM.tif]

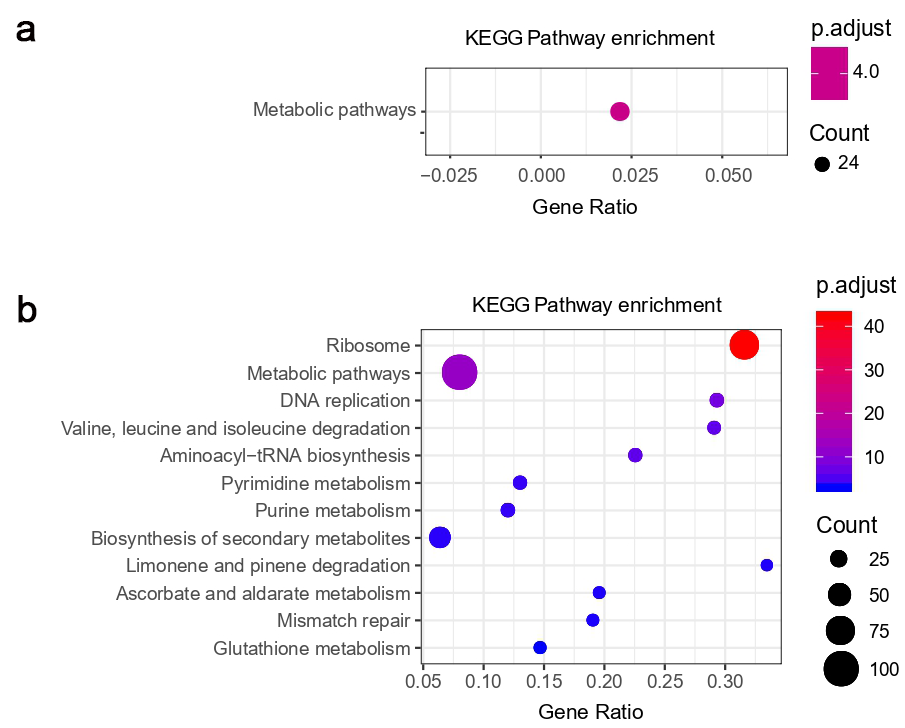

Supplement: Supplementary file 10 — Additional file 10: Figure S10. KEGG enrichment analysis of the DEGs in the axillary buds of genes that are respectively regulated by OEa lines (a), OEb lines (b) compared with the wild-type ZH11. Gene ratio indicates that the ratio of the DEG number and the number of genes has been annotated in this pathway. [file 12284_2020_446_MOESM10_ESM.tif]

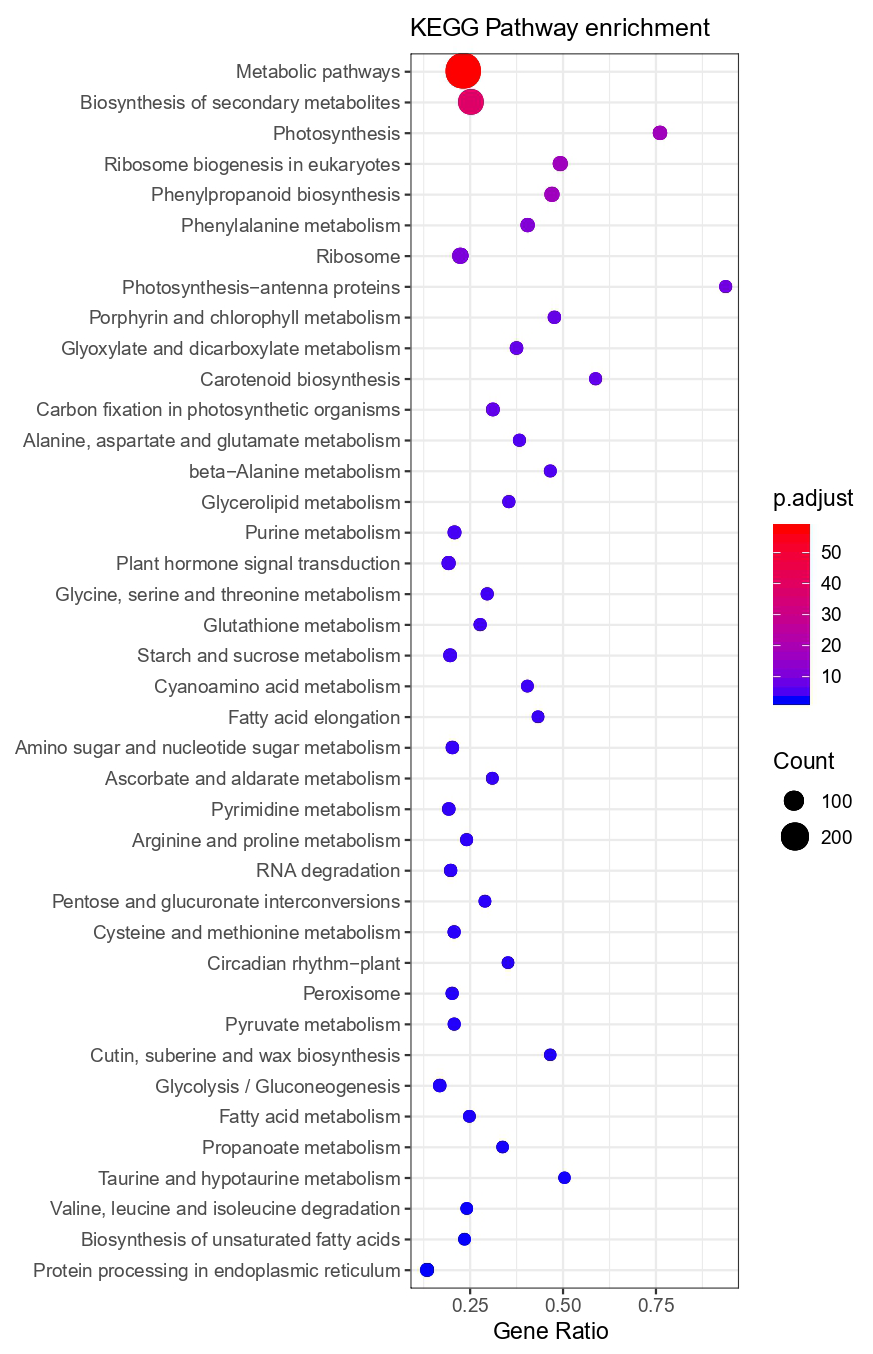

Supplement: Supplementary file 11 — Additional file 11: Figure S11. KEGG enrichment analysis of the DEGs in the axillary buds of genes that are jointly regulated by OEa and OEb lines compared with the wild-type ZH11. Gene ratio indicates that the ratio of the DEG number and the number of genes has been annotated in this pathway. [file 12284_2020_446_MOESM11_ESM.tif]

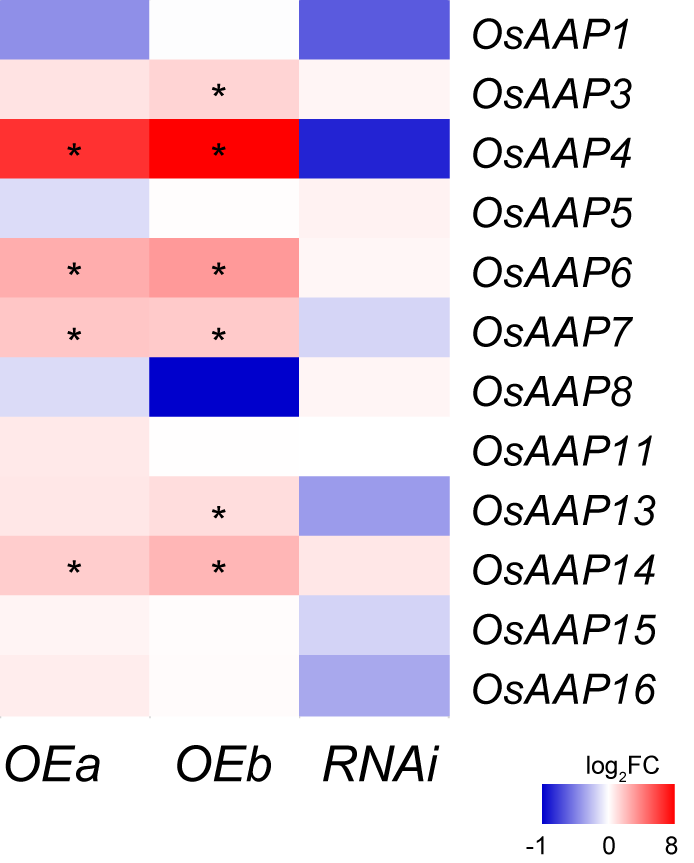

Supplement: Supplementary file 12 — Additional file 12: Figure S12. Heatmap visualization of expression profiles of DEGs in OsAAPs regulated by OsAAP4 OEa and OEb lines compared with the wild-type ZH11. Red boxes show up-regulation, and green boxes show down-regulation. “*” indicates a significant difference at P-value <0.05 and fold change >2. [file 12284_2020_446_MOESM12_ESM.tif]

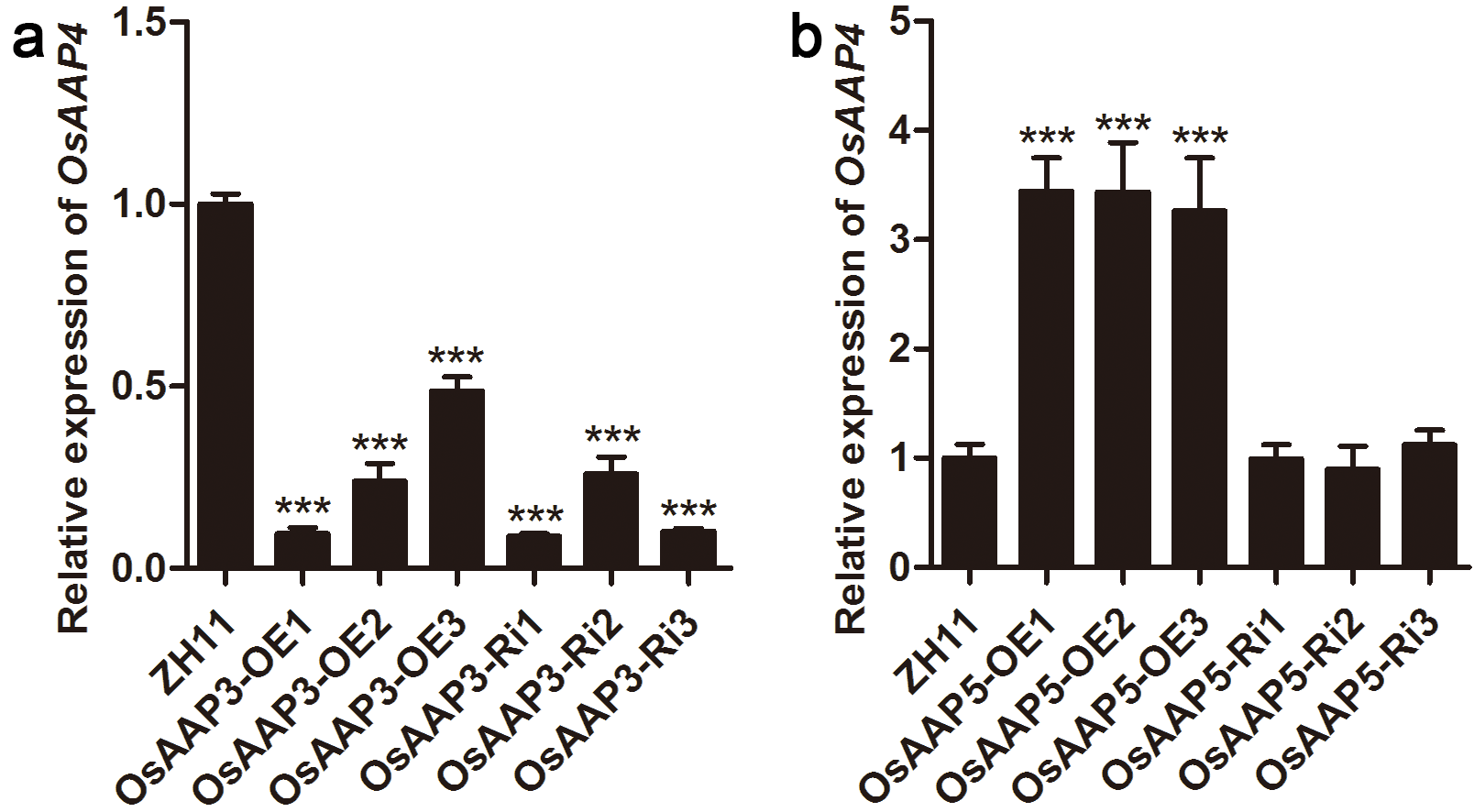

Supplement: Supplementary file 13 — Additional file 13: Figure S13. The expression of OsAAP4 in basal part of OsAAP3 and OsAAP5 transgenic plants. The primers used for quantifying OsAAP4 expression was F: GACATCGTCCACAACCTCAAGGCT, and R: GCCACAGCTCTAGCTAGGCAGC. The letters above the error bars are ranked by the T test, “***” indicates a significant difference at p < 0.001. Values are means ±SD (n = 3). [file 12284_2020_446_MOESM13_ESM.tif]
