# Supplementary material for: The Amino Acid Transporter OsAAP4 Contributes to Rice Tillering and Grain Yield by Regulating Neutral Amino Acid Allocation through Two Splicing Variants
Source: Rice (N Y). 2021 Jan 6;14:2. doi: 10.1186/s12284-020-00446-9 (PMC7788160; doi:10.1186/s12284-020-00446-9)
Supplement: Supplementary file 2 — Additional file 2: Figure S2. Sequencing of two different types of promoter sequences of OsAAP4. NIP indicates Nipponbare, C172 indicates the Hap5 type in Japonica. W144 indicates the Hap2 type in Indica. [file 12284_2020_446_MOESM2_ESM.docx]

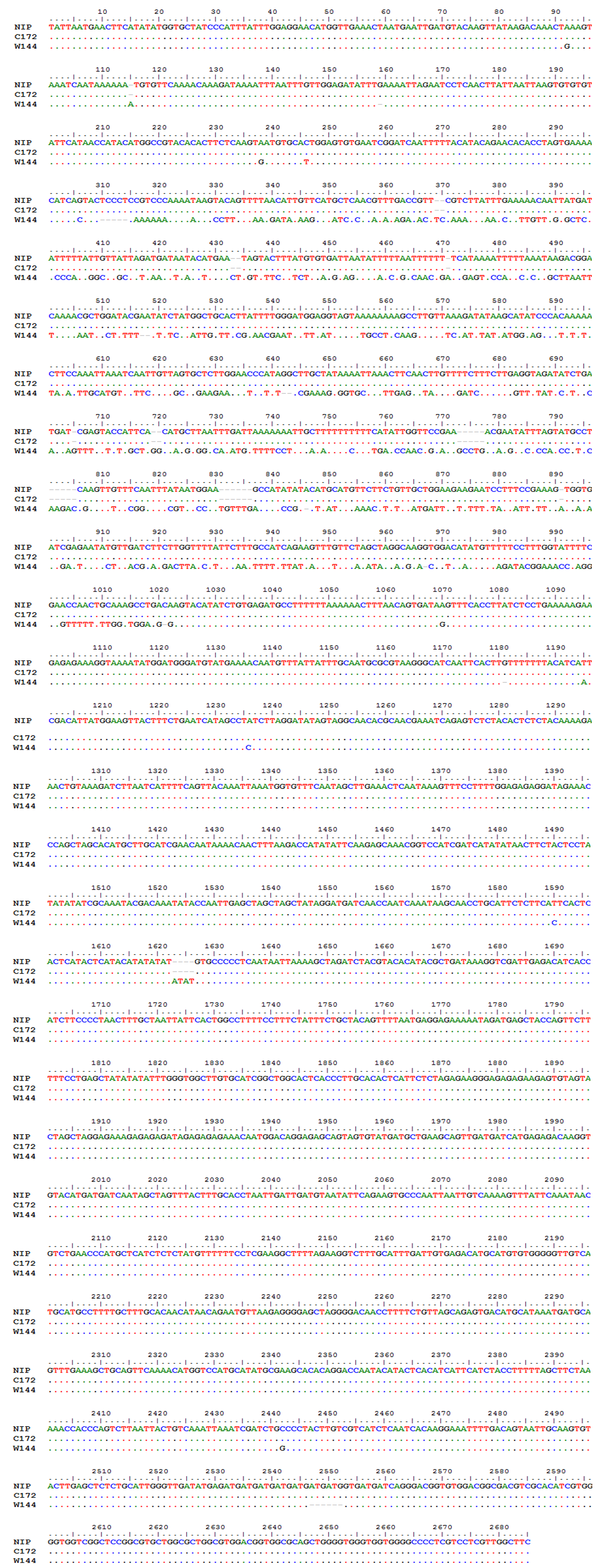


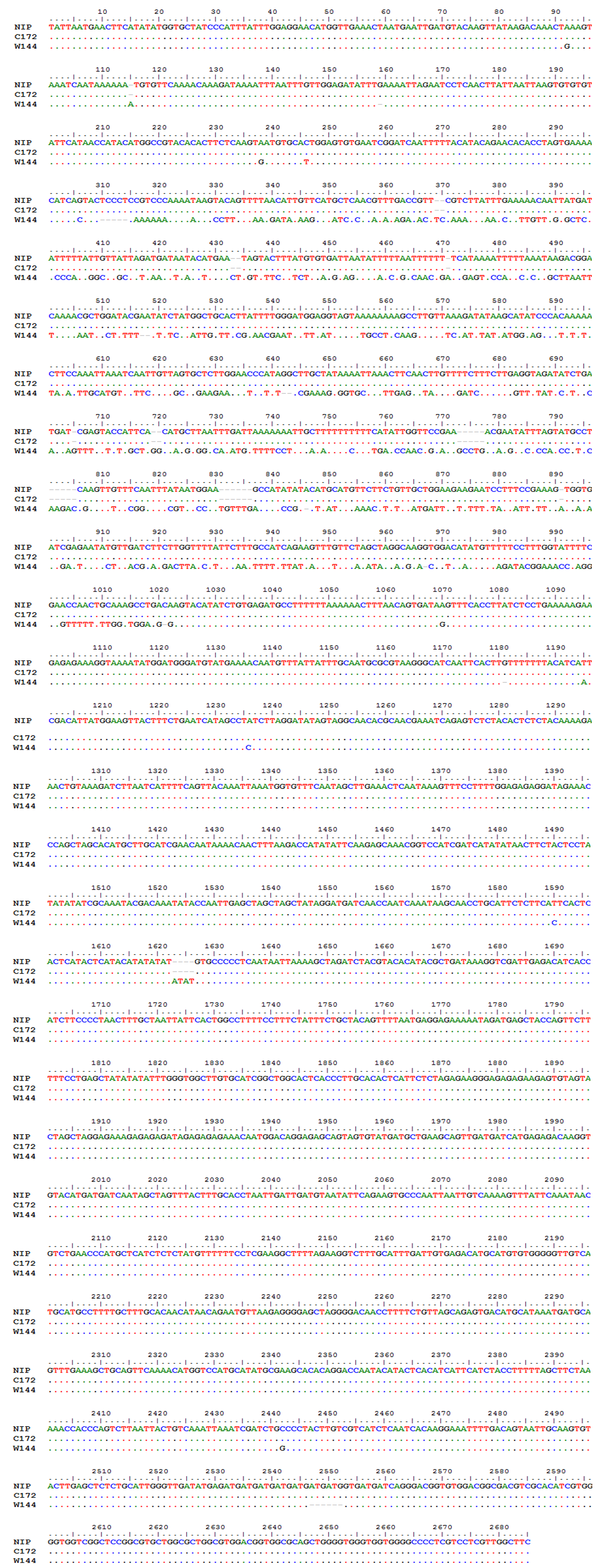


**Supplementary file 2: Fig. S2** Sequencing of two different types of promoter sequences of *OsAAP4*. NIP indicates *Nipponbare*, C172 indicates the Hap5 type in *Japonica*. W144 indicates the Hap2 type in *Indica*.
