# Supplementary material for: The Amino Acid Transporter OsAAP4 Contributes to Rice Tillering and Grain Yield by Regulating Neutral Amino Acid Allocation through Two Splicing Variants
Source: Rice (N Y). 2021 Jan 6;14:2. doi: 10.1186/s12284-020-00446-9 (PMC7788160; doi:10.1186/s12284-020-00446-9)
Supplement: Supplementary file 14 — Additional file 14 Table S1. List of the primers in this study. [file 12284_2020_446_MOESM14_ESM.docx]

Supplementary file 14: Table S1 List of the primers in this study.

| **Names** | **Forward sequence (5' - 3')** | **Reverse sequence (5' - 3')** |
| --- | --- | --- |
| p*OsAAP4-GUS* | TTAAGCTTATGGTGCTATCCCATTTATTTGGAGGA | TTGGATCCGCCGATGCACAAGCCACCCAA |
| *OsAAP4a*-GFP | GAAGATCTATGGACAGGAGAGCAGTAGTG | AAACTAGTGTTGACAGTCTTGAAGGGTGC |
| *OsAAP4b*-GFP | ATAGATCTATGATGATGATGATGATGATGGTGATGATCAG | ATACTAGTCGTAGTAGGTCGCGTTGATGA |
| *OsAAP4-*OEa | ATGGTACCATGGACAGGAGAGCAGTAGTG | ATTCTAGAGTTGACAGTCTTGAAGGGTGC |
| *OsAAP4-*OEb | ATGGTACCATGATGATGATGATGATGATGGTGATGATCAG | ATTCTAGACGTAGTAGGTCGCGTTGATGA |
| *OsAAP4-*RNAi1 | AGGATCCGAGACAAGGGACGGTGTGGA | AAGGTACCACCCACACAACATCACGTTCTTCG |
| *OsAAP4-*RNAi 2 | AGAGCTCGAGACAAGGGACGGTGTGGA | AAACTAGTACCCACACAACATCACGTTCTTCG |
| *OsAAP4*-U6IPST1 | GTGCTTCCACCGGGAAGGGTAGTTTTAGAGCTAGAAATAGCAAGTTA | TACCCTTCCCGGTGGAAGCACAACCTGAGCCTCAGCGCAGC |
| *OsAAP4*-U3IPST2 | ATGCTTCCACCGGGAAGGGTAGTTTTAGAGCTAGAAATAGCAAGTTA | TACCCTTCCCGGTGGAAGCATGCCACGGATCATCTGCACAACTC |
| q*OsActin* | CGGTGTCATGGTCGGAAT | GCTCGTTGTAGAAGGTGT |
| q*OsAAP4* | GACATCGTCCACAACCTCAAGGCT | GCCACAGCTCTAGCTAGGCAGC |
| q*OsAAP4a* | TGGCACTCACCCTTGCACAC | CCGTCCACACCGTCCCTTGT |
| q*OsAAP4b* | ACTTGAGCTCTCTGCATTGGGT | AGCGGTAGCAATTGGCGAGGA |
| q*OsAAP4b+c* | TTGCTGCAGGTGTTCGCGCA | ATCGTCCGCAGCACCAGCTTCAG |
